# Wetting of Superhydrophobic Polylactic Acid Micropillared Patterns

**DOI:** 10.1021/acs.langmuir.2c01708

**Published:** 2022-08-05

**Authors:** Eda Hazal Tümer, H. Yildirim Erbil, Numan Akdoǧan

**Affiliations:** † Department of Chemical Engineering, Gebze Technical University, Gebze, Kocaeli 41400, Türkiye; ‡ Department of Physics, Gebze Technical University, Gebze, Kocaeli 41400, Türkiye

## Abstract

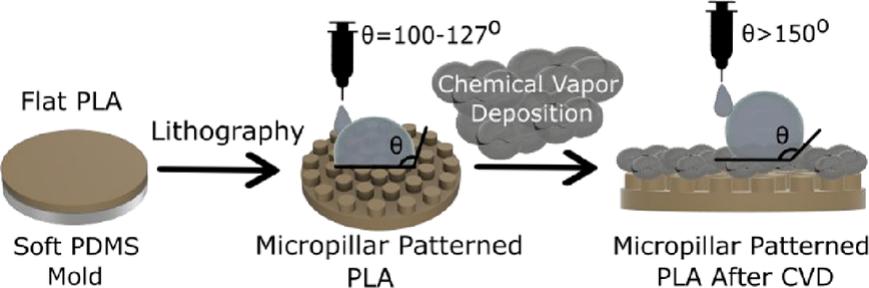

Superhydrophobic (SH) polylactic acid (PLA) surfaces were previously produced by various methods and used especially in biomedical applications and oil/water separation processes after 2008. However, the wettability of SH-PLA patterns containing micropillars has not been investigated before. In this study, PLA patterns having regular microstructured pillars with 12 different pillar diameters and pillar-to-pillar distances were prepared by hot pressing pre-flattened PLA sheets onto preformed polydimethylsiloxane (PDMS) soft molds having micro-sized pits. PDMS templates were previously prepared by photolithography using SU-8 molds. Apparent, advancing, and receding water contact angle measurements were carried out on the PLA patterns containing micropillars, and the morphology of the patterns was examined by optical and SEM microscopy. The largest contact angle obtained without the surface modification of the pure PLA pattern was 139°. Then, PLA micropatterns were hydrophobized using three types of silanes via chemical vapor deposition method, and SH-PLA patterns were obtained having θs of up to 167°. It was found that the highest θ values could be obtained when PLA pattern samples were coated with a silane containing a fluorine atom in its chemical structure. Washing and service life stability tests were also performed on the coated pattern samples and all of the silane coatings on the PLA patterns were found to be resistant over a 6 month period.

## Introduction

Poly(lactic acid) or polylactide (both of them are abbreviated as PLA) is a biocompatible, recyclable, biodegradable, and environmentally friendly polymer. It is mostly produced from renewable sources such as corn, sugar cane, etc., where large amounts of CO_2_ gas is consumed during the process.^1−10^ PLA has the potential to be used in biomedical applications since it is biocompatible with body fluids, and its degradation products does not interfere with tissue healing, where the obtained metabolites are incorporated into the tricarboxylic acid cycle after hydrolysis and can be excreted from the body.^4,10−12^ The PLA surface is relatively hydrophobic, having a static water contact angle in the range of 75–85°, and has a low cell affinity in medical applications.^13,14^ Implants and fracture fixation devices for the healing of tendons and ligaments are produced using PLA to replace metal-based medical devices. The good thermal processability of PLA allows its use in injection molding, film extrusion, blow molding, thermoforming, 3D printing, fiber spinning, and other film-forming applications. It has also flame retardancy and weather resistance properties.^1,2,7−9^ Very recently, ultrastrong, super-tough, and thermally stable PLA/nucleating agent composites were prepared, giving a chain-like interlocking shish-kebab structure with gradients in both length and thickness.^15^ The slow degradation rate of PLA is a problem for the disposal of PLA packaging films, and a large amount of research was devoted to speeding up the PLA decay rate in order to substitute the conventional packaging films with PLA as an environmental friendly bioplastic.^1−7,9^ On the other hand, pure PLA or composite PLA filaments blended with suitable polymers are one of the most widely used raw materials in the “fused deposition modeling” (FDM)-based 3D printing process due to its ease of use and a wide color range.^16−19^ Controlling the wettability of PLA surfaces is an important process in both academic and industrial studies.

The wettability of a solid can be quantified by the measurement of the contact angle (θ) of a liquid drop on it. The θ value depends on the magnitude of intermolecular interactions between the contacting liquid and the substrate. θ is the observed angle between the tangent to the solid surface and the tangent to the liquid–fluid interface at the contact line between the three phases.^20−22^ There are three types of θ. An “apparent contact angle” represents the “average” θ for the entire three-phase contact line of a drop and can be measured after rapidly removing the needle from the drop placed on a solid. An “advancing contact angle”, θ_a_, is measured when the volume of the drop is increased through the needle and the three-phase contact line advances on a fresh solid surface. A maximum value of θ_a_ is reached before the three-phase line is broken. A “receding contact angle”, θ_r_, is measured when the volume of a previously formed sessile drop on the substrate is reduced by applying suction to a portion of liquid from the drop through a needle. θ_r_ represents the minimum value of contact angle before the three-phase line is broken, which is used to describe the strength of liquid/solid adhesion. The difference between θ_a_ and θ_r_ gives the “contact angle hysteresis” or the CAH value, which is a measure of the surface roughness and chemical heterogeneity of the substrate.^20−22^ Solids are generally defined as “hydrophilic” and “hydrophobic”. Hydrophilic surfaces have apparent water contact angles less than 90°, and hydrophobic surfaces have θs of ≥90°. Surfaces having water at θ > 150° are called “superhydrophobic” (SH). Water droplets can roll off easily on a SH surface at a roll-off angle (tilt angle) of less than 10° at ambient conditions.^23−26^

Synthetic water-repellent SH surfaces were developed via biomimicking examples in nature. A lotus leaf has SH properties and repels dirt and mud due to the specific dual size range roughness on its surface. Both micrometer-scale papillae (5–9 μm in diameter) and nanosized protrusions with average diameters of 124 ± 3 nm are present on the lotus leaf surface, which are covered with an epicuticular wax to provide low surface-free energy. The surface structure of the lotus leaf creates air trapping when in contact with water droplets and results in very high water contact angles that are larger than 150°.^27^ Many other plants and animals such as rice leaves, butterfly wings, mosquito eyes, cicada wings, and gecko feet exhibit similar SH characteristics.^28^ Synthetic SH surfaces can be produced by introducing nano- and/or microscale roughness on a hydrophobic substance having low surface free energy or, alternatively, by coating a preformed micro-/nanostructured rough surface with a thin hydrophobic layer having low surface free energy.^24−26,28,29^ SH surfaces have the potential to be used in industry as self-cleaning exterior paints, transparent windows, windshield glass, car bodies, mirrors, dirt- and dust-repellent solar energy panels, surveillance cameras, sensors, lenses, telescopes in optical industry, and so on.^26,28−34^

Many approaches were developed to model the wetting behavior of solid surfaces having different chemical groups and roughnesses by forming patterns containing pillars and/or pits. Contact angle studies on the solids containing periodic micro-/nanopatterns, which were obtained mainly by photolithography and other methods such as e-beam, X-ray lithography, and etching, are an important branch of wetting studies.^35−42^ The chemical vapor deposition (CVD) method was frequently applied to coat thin films on the hydrophilic or only partially hydrophobic patterned surfaces to impart superhydrophobicity. Atmospheric CVD has some advantages over other methods since it does not need a vacuum, and only a small amount of chemicals are consumed during evaporation and condensation on the substrates to form a thin homogeneous coating.^43^

PLA polymer was used to produce a SH surface for the first time in 2008.^44^ The authors adapted the phase inversion method for this purpose, which was formerly used to produce a SH polypropylene surface.^23^ They dissolved 5–12.5% (w/v) PLA polymer concentrations in dioxane solvent at room temperature, and water or ethanol were used as non-solvents. Thin membranes having SH property were obtained after spreading the polymer solutions onto clean glass Petri dishes and dried under air overnight, followed by vacuum drying for 48 h at 40 °C.^44^ In the same study, cooling the solution-cast film down to −20 °C was also applied to control the resultant surface structure of SH-PLA. Water θ increased from 67 to 153° on the sponge-like porous film, which was obtained by applying a gelation-in-air procedure of PLA in dioxane followed by precipitation in ethanol. It was reported that the addition of a non-solvent was not useful for the increase of the water θ.^44^

Such water repellent SH-PLA surfaces were later used in biomedical applications, and they prevent the adhesion and proliferation of bone marrow-derived cells that were previously isolated from the femurs of rats in comparison with smooth PLA surfaces prepared by simple solvent casting.^45^ In another study, chloroform and dichloromethane were used as solvents for the PLA, and absolute ethanol, *n*-butyl alcohol, and *n*-butyl acetate served as non-solvents where the resultant multiscaled SH-PLA surfaces on glass exhibited water θs of up to 156°.^46^ The same group prepared SH-PLA surfaces using solvent/ternary non-solvent-coated PLA films to control the adhesion of water.^47,48^ Only chloroform was used as the solvent, and *n*-butyl acetate, absolute ethanol, and *n*-butyl alcohol were mixed together at equal volume ratios to be used as the ternary non-solvent. The obtained polymer solution was coated onto another flat PLA substrate to form an SH-PLA layer and used to form microdroplet arrays in biochips for a no-mass-loss transport process.^47,48^ The addition of nanosilica particles into the PLA polymer solution was tried, and water θ increased up to 167° for the final composite PLA film, giving a porous network structure after phase inversion.^49,50^ Alternatively, the addition of poly-d-lactic acid (PDLA) polymer to the poly(l-lactic acid) (PLLA) polymer resulted in the formation of stereo-complexed crystals, giving a morphology that was very different from the porous structure of pure PLLA with a water θ up to 155° and having good anti-icing properties.^51^ A SH-PLA foam to be used for oil–water separation was prepared after dissolving PLA in dioxane solvent and applying freeze-drying and skin peeling. This PLA foam with a water θ of 151° can absorb oil 32 times its own weight through the micro- and nanostructures on its surface.^52^ Recently, a novel microsphere-modified non-woven polyester fabric has been produced, which was coated with PLA using dip-coating in a triphasic solution. The produced fabric was successfully used for oil–water separation with a high separation efficiency of over 97% after 10 cycles.^53^

Superhydrophobicity was also introduced onto nonwoven and woven PLA fabrics by the addition of suitable nanoparticles to the PLA polymer^54−58^ or hydrophobization with silanes.^59,60^ These SH products were especially used in oil/water separation applications. PLA objects with large surface patterns in the mm size range were prepared using a 3D printer, and later, PLA filaments and hydrophobic coatings with nanoscale structures were formed on these surfaces by a dip-coating process using a dispersion of hydrophobic silica nanoparticles in methyl ethyl ketone solvent, giving a SH-PLA surface where the water θ reached >155°.^61^ Solvent etching and nanoparticle decoration methods were also applied to produce SH-PLA for oil–water separation utilization.^62,63^ For example, a 3D-printed PLA porous membrane was etched in acetone to form peony-like microstructures and then immersed in dopamine and decorated with polystyrene nanospheres to obtain a final SH-PLA membrane. A high oil–water separation efficiency and high liquid flux was obtained with this membrane.^62^ In another study, packing materials made of SH-PLA were used to separate hexane solvent that was present in a water emulsion.^63^

The electrospinning of PLA was also used to obtain the SH-PLA mats.^64−68^ A non-woven nanofibrous SH-PLA Janus fabric was produced via an electrospinning technique on a cotton substrate and used in an oil/solvent–water separation process.^64^ An SH-PLA composite membrane containing magnetic γ-Fe_2_O_3_ nanoparticles was formed via electrospinning and exhibited good mechanical resistance, anti-icing performance, high oil adsorption capacity, and high oil permeation flux.^65^ In another study, PLA was electrospun from its dichloromethane–dimethyl formamide solution, and its surface was modified by coating with the titanium dioxide nanoparticles and later with polysiloxane to reduce the final surface free energy. This SH-PLA composite membrane exhibited high permeation flux and good filtration efficiency in the oil–water separation process, where toxic methylene blue could be adsorbed rapidly from an aqueous solution.^66^ Glycerol was also employed to provide −OH functional groups in the PLA matrix during the electrospinning of SH-PLA nanofibers, and the obtained fiber mat was then immersed in alkyl ketene dimer solution to increase its hydrophobicity by a grafting reaction to impart oleophilicity. This fiber mat showed high oil absorption rates and capacities.^67^ Electrospun composite SH-PLA nanofibers were also used in biomedicine to protect wounds from bacteria or organisms and promote skin regeneration. For this purpose, PLA + poly(vinyl pyrrolidone)/PLA + poly(ethylene glycol) core/shell fibers were produced by electrospinning and loaded with bioactive agents. This mat inhibited adhesion and spreading of the exogenous bacteria and led to faster healing of burns than a conventional product.^68^

The surface modification of PLA is another important field in modifying the chemical structure of the top layers. For this purpose, methods such as surface alkaline hydrolysis treatment, graft polymerization, plasma treatment, and various surface chemical reactions are used.^17,69−72^ Plasma surface modification of PLA was reported several times in the literature; however, the gains achieved with this method are partially lost due to degradation and surface chemical group rearrangements when the macromolecular motions are thermally activated to minimize the interfacial energy.^73,74^ Silane or siloxane coatings on PLA were also successfully applied; for example, organosilanes such as (3-aminopropyl)triethoxy silane, and (3-glycidoxypropyl)-trimethoxy silane were coated on PLA after oxygen plasma application.^17,75,76^

Although SH-PLA surfaces were prepared and characterized more than a decade ago, no article was published on the wettability of SH-PLA surfaces having regular pillar-type patterns on it. It is well known that the investigation of contact angles and the evaluation of the presence of Cassie and Wenzel states or the transition from Cassie to Wenzel states on micro- or nanostructured SH surfaces is an important field to evaluate the suitability of a material for various industrial and academic applications.^22,35−42,77−79^ In order to fill this gap, we prepared microstructured pillar-type PLA surfaces by hot-pressing previously flattened PLA sheets that were prepared by 3D-printing onto the preformed polydimethylsiloxane (PDMS) templates having microsized pits with 12 different diameters and separation distances in this study. PDMS templates were prepared by soft lithography using a negative pattern of pillared SU-8, which was prepared by photolithography. Apparent, advancing, and receding water θ measurements were made on the PLA patterns containing regular pillars, and optical microscopy and scanning electron microscopy (SEM) were applied to examine the morphology of the patterns. Since a SH-PLA pattern could not be obtained directly without surface modification (water θ < 139° on a pure PLA pattern), surface hydrophobizations were also applied to get the SH-PLA patterns having θs of >150°. For this purpose, three different silanes, dimethyldichlorosilane (DMDCS), *n*-propyltrichlorosilane (NPTS), and (tridecafluoro-1,1,2,2-tetrahydrooctyl) trichlorosilane (TDFS) were coated onto the PLA patterns by the CVD method. The wettability on the patterns with pillars made of SU-8 with micropits made of PDMS and with pillars made of PLA was also investigated in this study, and the results were discussed in comparison with the previously published data for SU-8 and PDMS patterns.

## Experimental Section

### Materials

“Makerbot Replicator Mini+” 3D printer and “Makerbot” commercial PLA filaments were used to obtain the PLA sheets having a thickness of 0.5 mm. “SU-8 2015” is an epoxy-based negative photoresist supplied by Kayaku Chemicals. “Mr-Dev-600” is the developer that was used to remove the unexposed SU-8 photoresist and is supplied by “Micro-Resist Technology GmbH”. PDMS (Sylgard 184 silicone elastomer and curing agent) is supplied by Dow Silanes. DMDCS (Fluka), NPTS (Acros Organics), and TDFS (ABCR) were used as received for the surface modification of PLA patterns. A UV-EX Pioneer Lithography System (Mikronya Lithography Systems Ltd.) and a “PTL-VM500” vacuum plasma device, which both are present in Gebze Technical University, Microfluidics Chip Laboratory, were used to carry out the SU-8 photolithography process.

### Methods

**Figure 1:**
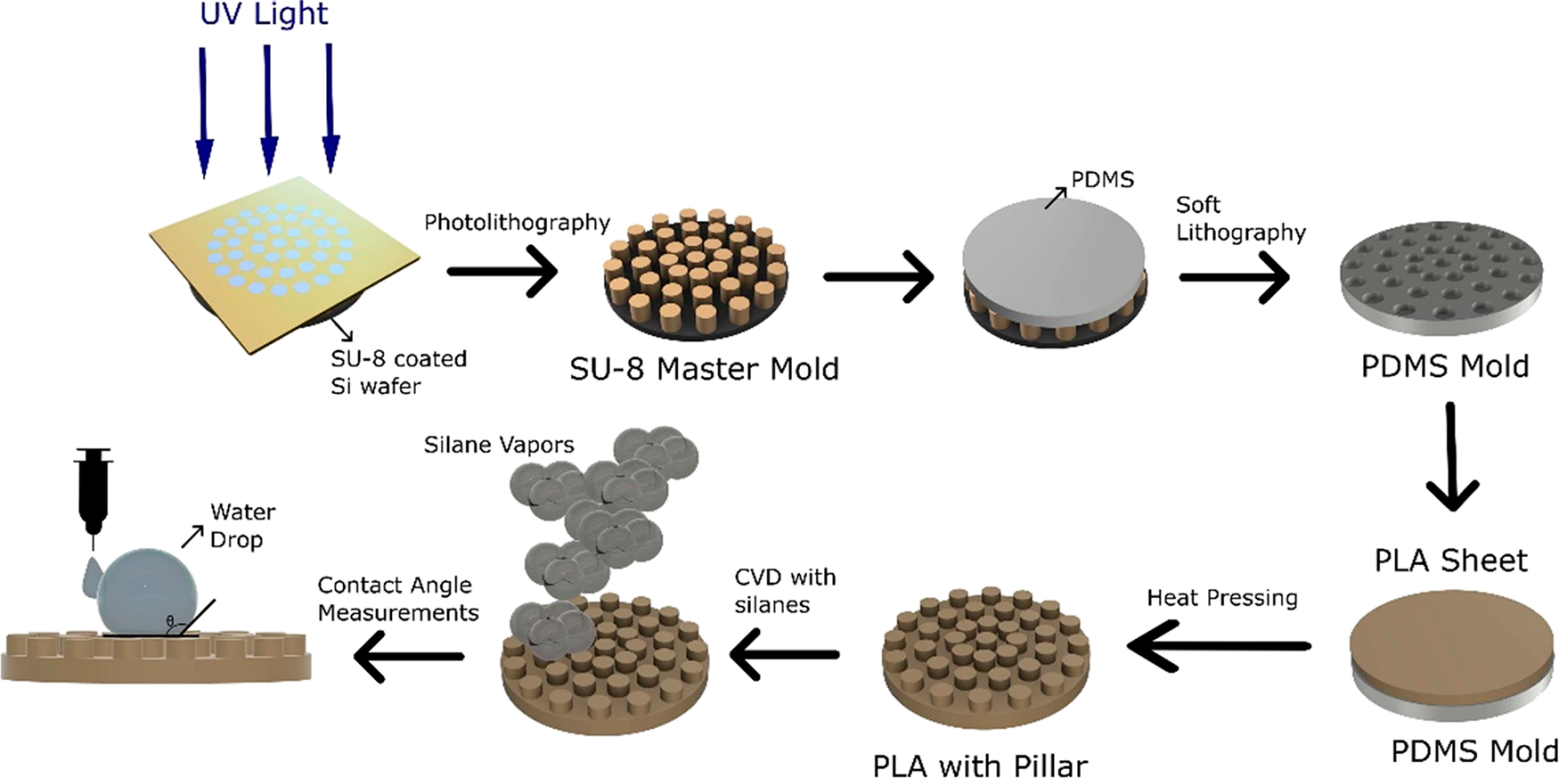
Schematic description of the seven steps that were carried out for the preparation of superhydrophobic PLA micropillared patterns.

There were seven steps to obtain a pillared SH-PLA pattern: Designing and printing the chrome mask, preparation of the pillared SU-8 template, preparation of the PDMS template containing micropits, 3D printing of the thin PLA sheet, surface flattening of the PLA sheet, patterning the PLA sheet by hot-pressing it onto the PDMS template, and surface modification of the PLA pattern by the CVD of three silanes at room temperature. The description of the seven steps carried out for the preparation of superhydrophobic PLA micropillared patterns is given schematically in Figure 1.

#### Chrome Mask Design and Printing

The chrome mask to obtain cylindrical pillars was designed using Clewin-5 software. Twelve different combinations of pillar diameters and pillar-to-pillar distances were designed to fit a 4 inch diameter circular space. The dimensions of the patterns are given in Table 1. Lua programming language, which was supported by the Clewin-5 software, was used for the preparation of the circular designs as given in Figure S1 of the Supporting Information. The designed mask was printed with the help of a mask writer at Bilkent University, UNAM on a commercial 5″ photomask with a 100 nm chromium layer on a soda lime supplied from Nanofilm company.

**Table 1:** Sample Codes and the Dimensions of the Pillars

sample name	pillar diameter (μm)	pillar-to-pillar distance (μm)
dia10-dis10	10	10
dia10-dis15	10	15
dia10-dis20	10	20
dia10-dis25	10	25
dia15-dis15	15	15
dia15-dis20	15	20
dia15-dis25	15	25
dia20-dis20	20	20
dia20-dis25	20	25
dia20-dis30	20	30
dia25-dis25	25	25
dia40-dis40	40	40

#### Preparation of the SU-8 Patterns

A 4 inch Si wafer was kept in acetone for 3 min and isopropyl alcohol (IPA) for 3 min in an ultrasonic bath for surface cleaning and dried while keeping it on an electric heater at 95 °C for 5 min. Oxygen plasma was then applied for 2 min around ∼10^–1^ mbar at room temperature. Then, SU-8 coating was applied using a spin coater according to a recipe that was suggested in the SU-8 2015 datasheet. In the first step, a rotating speed of 500 rpm was applied by incrementally increasing the speed in 100 rpm steps and finally rotating at 500 rpm for 5 s. In the second step, a rotating speed of 2000 rpm was applied for 30 s in increments of 300 rpm, and the SU-8 coating process was completed. Then, the SU-8-coated Si wafer was kept on a heater at 65 °C for 1 min during the prebake stage to avoid possible cracks on the SU-8 layer. The SU-8-coated Si wafer was kept on the heater at 95 °C for 3 min for the soft bake stage. Afterward, an exposure energy of 147 mJ/cm^2^ (49 mW/cm^2^ power for 3 s) was applied by the UV light source (365 nm) through the prepared mask for the curing of the SU-8 layer. After the curing, the SU-8-coated Si wafer was kept on the heater at 65 °C for 1 min and then at 95 °C for 2 min for the post-exposure bake stage to complete the photoreaction that begins during the UV exposure. Later, the uncured SU-8 polymer was removed using Mr-Dev-600 developer by keeping the SU-8-coated Si-wafer in the developer for 3 min. Samples were then washed with IPA. When a turbidity (white film) appeared on the surface, it was immersed back into the developer and left for one more minute. After the IPA washing, the sample was again kept in acetone for 1 min, washed rapidly with IPA, and dried under nitrogen gas flow. The purpose of the last washing in IPA is to prevent the remaining acetone from staining the SU-8 coating. Finally, the samples were annealed on a heater at 95 °C for 3 min to complete the production of the pillared SU-8 master templates.

#### Preparation of the PDMS Patterns

PDMS-negative templates containing cylindrical micropits are needed to obtain PLA pillars by hot pressing. SU-8 patterns having cylindrical pillars were used as negative templates to prepare the PDMS patterns. SU-8 pattern surfaces were coated with trimethylchlorosilane by the CVD method for 40 min at room temperature to decrease the possible adhesion between SU-8 and PDMS before pouring the mixture of PDMS and curing agent. PDMS and curing agent (10:1 w/w) were mixed until the required viscosity was reached, and the mixture was kept in a vacuum desiccator for approximately 40 min at room temperature to remove the small air bubbles. Later, PDMS mixture was poured onto the pillared SU-8 pattern and kept on a heater at 95 °C for 30 min. After cooling to room temperature, the negative PDMS soft pattern and SU-8 template could be easily separated due to the presence of the trimethylchlorosilane coating on the SU-8 pattern.

#### 3D-Printing of the PLA Sheet

A thin circular 3D model of the PLA sheet was drawn in Fusion 360 with Makerbot Print software, and the PLA sheets in white color with a diameter of ∼9 cm and a thickness of 0.5 mm were printed with a Makerbot Replicator Mini+ 3D printer using commercial PLA filaments of Makerbot.

#### Surface Flattening of the PLA Sheet

**Figure 2:**
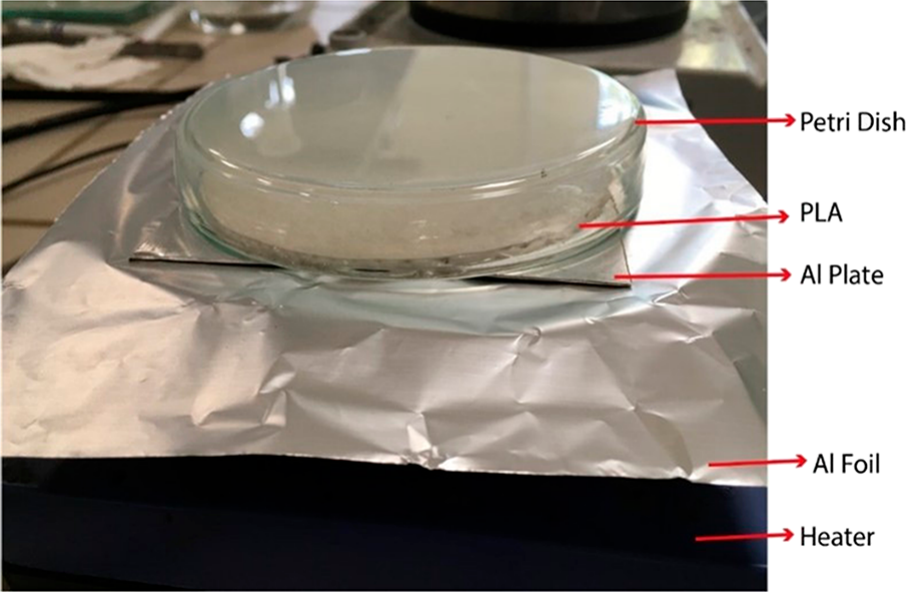
Surface flattening of the PLA sheet by heat application.

Since the 3D printed circular PLA sheet had considerable surface roughness, it was not suitable for patterning directly with hot-pressing. Thus, the surface flattening of the PLA sheet was carried out by applying heat as seen in Figure 2. The temperature of the hot plate heater was set to 140 °C, and an aluminum plate was located on the heater to prevent the adhesion of PLA to the heater plate after softening. A Petri dish was placed on the PLA sheet to ensure that the applied heat would remain on the PLA surface and the effect of the temperature changes in the environment would be minimized. After keeping on the heater for 3 min, the PLA sheet was left to cool slowly to room temperature in 8–10 min to avoid ripples on the PLA surface due polymer shrinking.

#### Patterning the Flat PLA Sheet by Hot Pressing it on the PDMS Pattern

The flattened surface of the PLA sheet was placed on the PDMS template containing micropits upside down in a Petri dish. The cover was closed, and this setup was placed in a vacuum oven where full vacuum (approximately 0 mbar) was applied from the laboratory line. The temperature of the oven was increased to 190 °C in 60 min and kept at this temperature for 30 min. This assembly was then removed from the vacuum oven and allowed to cool to room temperature in about 30 min. Afterward, the patterned PLA was slowly separated from the PDMS pattern. Figure S2 of the Supporting Information shows the photo of the pillared PLA sheet after the pattern formation.

#### Surface Modification of PLA Patterns with Silanes by Applying the CVD Method

Since a water apparent θ of 150° could not be obtained on the pillared PLA pattern samples, the surface hydrophobization of the PLA patterns were carried out at 65 °C using three types of silanes to obtain a pillared SH-PLA pattern. Only DMDCS was dissolved in hexane (1:1), and TDFS and NPTS were used directly. The properties of the used silanes are given in Table S1 of the Supporting Information. Then, PLA patterns were kept in a desiccator overnight where 5 mL of the silane liquids were present in a glass cup (10 mL for DMDCS). Afterward, the silane-coated PLA patterns were cleaned with ethanol, 50:50 ethanol–water mixture, and pure water, respectively. Then, they were dried in a vacuum oven at 50 °C for 2 h. Gravimetric analysis was applied after CVD deposition, and the percentages of the silanes that were held on the PLA surface are given in Table 2.

**Table 2:** Apparent, Advancing, and Receding Water θ Results on the Flat and Pillared SU-8 Patterns Having 20 ± 1 μm Height

sample name	apparent θ (°) ± 1	θ_a_ (°) ± 1	θ_r_ (°) ± 2	CAH (°) ± 2
flat SU-8	80	90	33	57
dia10-dis10	137	155	88	67
dia10-dis15	131	153	89	64
dia10-dis20	122	127	65	62
dia10-dis25	115	116	58	58
dia15-dis15	126	137	80	57
dia15-dis20	128	140	77	63
dia15-dis25	111	114	58	56
dia20-dis20	134	142	104	37
dia20-dis25	124	130	73	57
dia20-dis30	114	117	58	59
dia25-dis25	120	123	65	58
dia40-dis40	112	118	69	49

## Results and Discussion

In this section, the optical microscopy and scanning electron microscopy (SEM) images of the patterned samples were presented. Advancing and receding water θ results for the pillared SU-8 patterns, PDMS patterns containing micropits, and pillared PLA patterns were also given. Finally, the PLA patterns after surface hydrophobization by DMDCS, TDFS, and NPTS using the CVD methods were examined and discussed.

### Pillared SU-8 Patterns

**Figure 3:**
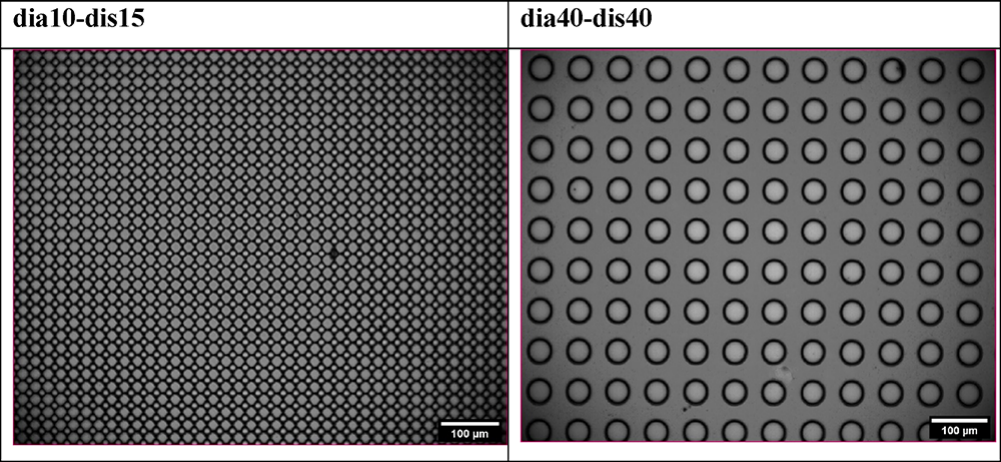
Indicative optical microscope images of two different pillared SU-8 pattern samples.

Two indicative optical microscope images of the pillared SU-8 patterns are given in Figure 3. Optical microscopy images of all pillared SU-8 patterns are provided in Figure S3 of the Supplementary Information.

Indicative images of water droplet profiles on the SU-8 patterns are given in Figure S4 of the Supporting Information. The apparent, advancing, and receding water θ results on the pillared SU-8 patterns having a height of 20 ± 1 μm are given in Table 2.

The results given in Table 2 suggest that large θ_a_ (153–155°) and θ_r_ (88 and 89°) values were measured on the SU-8 patterns made of small pillars and short separation distances as seen for (dia10-dis10) and (dia10-dis15) samples. Conversely, small θ_a_ (117–130°) and θ_r_ (58–73°) values were measured for the SU-8 patterns made of large pillars and long separation distances as seen for (dia20-dis25), (dia20-dis30), (dia25-dis25), and (dia40-dis40) samples. The increase in separation distance for the constant pillar diameter case caused a decrease in all type of the contact angles similar to the previously published results on the pillared SU-8 patterns.^80,81^

In general, there was a moderate effect of the change in the SU-8 pillar dimensions on the measured contact angles and the average apparent θ was equal to 123 ± 13°, where θ_a_ = 131 ± 20°, θ_r_ = 67 ± 23°, and CAH = 57 ± 9° (except the dia20-dis20 sample). The average deviations were around 15% for both apparent θ and θ_a_ values. In addition, the apparent θ, θ_a_, and θ_r_ results for (dia15-dis15), (dia25-dis25), and (dia40-dis40) pillared SU-8 patterns and the same-sized samples given in ref (80) having pillar heights of 30 μm were compared in Table 3.

**Table 3:** Comparison of the Apparent, Advancing, and Receding Contact Angles and CAH Values Obtained for SU-8 Patterns in this Study and the Results Reported in ref (80)

	ref^80^				present study			
sample name	apparent θ (°)	θ_a_ (°)	θ_r_ (°)	CAH (°)	apparent θ (°) ± 1	θ_a_ (°) ± 1	θ_r_ (°) ± 2	CAH (°) ± 2
dia15- dis15	145	152	99	53	126	137	80	57
dia25- dis25	146	153	105	48	120	123	65	58
dia40- dis40	145	152	97	55	112	118	69	49

As seen in Table 3, the apparent θ, θ_a_, and θ_r_ values in this study were around 26 ± 10° less than that of given in ref (80). However, the CAH values (53 ± 4°) were very close (within 8%). The small θ values for the SU-8 patterns can be explained by the short pillar heights used in the present study (20 ± 1 μm), which was around 10 μm shorter than that of the pillar height reported in ref (80). The apparent θ values of 98–102° reported for the SU-8 samples having pillar heights of 12–13 μm in ref (80) supports this conclusion.

For the apparent θ on the flat SU-8 layer, a value of 80° was measured as given in Table 2, which well fits the literature value where an apparent θ of 79 ± 1° was reported on a flat SU-8 layer.^82^ Then, the contact angle value on the flat SU-8 was used to calculate the theoretical contact angles on pattern surfaces by using Wenzel^83^ and Cassie–Baxter equations^84^ as given in Table S2 of the Supporting Information, and it was found that none of the contact angle results on the SU-8 patterns fit these equations as expected.^22,26,77−81^

### PDMS Patterns Containing Micropit Arrays

**Figure 4:**
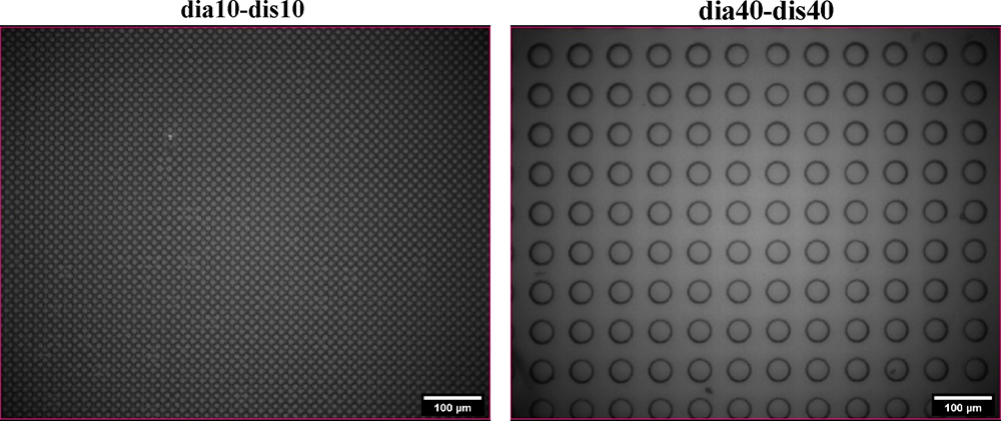
Indicative optical microscope images of two different PDMS soft patterns containing cylindrical micropits.

Two indicative optical microscope images of the PDMS soft pattern samples containing cylindrical micropits are given in Figure 4. All the optical images of the PDMS soft patterns are given in Figure S5 of the Supporting Information. SEM images of the PDMS pattern samples of (dia10-dis10) in 250×, 500×, and 1000× magnifications are given in Figure S6, and those of the (dia40-dis40) pattern sample in the same magnifications are given in Figure S7 of the Supporting Information. Uniform cylindrical micropits were seen in all of these images with diameters and pit-to-pit separation distances fitting the nominal values and having an average depth of 18 ± 2 μm.

Indicative images of water droplet profiles on the PDMS micropit patterns are given in Figure S8 of the Supporting Information. The apparent, advancing, and receding water θ results on the PDMS patterns containing micropit arrays are given in Table 4.

**Table 4:** Apparent, Advancing, and Receding Water θ Results on the Flat PDMS Layer and on the PDMS Patterns Containing Micropit Arrays with Depth of 19 ± 1 μm

sample name	apparent θ (°) ± 1	θ_a_ (°) ± 1	θ_r_ (°) ± 2	CAH (°) ± 2
flat PDMS	108	119	95	24
dia10-dis10	143	157	97	60
dia10-dis15	124	132	104	28
dia10-dis20	120	131	78	53
dia10-dis25	124	133	65	68
dia15-dis15	145	130	101	28
dia15-dis20	124	141	81	59
dia15-dis25	121	132	93	39
dia20-dis20	131	137	99	38
dia20-dis25	122	137	83	54
dia20-dis30	122	129	89	40
dia25-dis25	122	144	100	45
dia40-dis40	121	130	75	55

The results given in Table 4 suggested that large θ_a_ (144–157°) and θ_r_ (97–100°) values were measured on the PDMS patterns having micro-pits, where the pit diameters were equal to separation distances as seen for (dia10-dis10) and (dia25-dis25) sample results. Conversely, small θ_a_ (129–133°) and θ_r_ (65–89°) values were measured for the PDMS patterns, where the separation distances were longer than diameters as seen for (dia10-dis25), (dia15-dis25) and (dia20-dis30) sample results. The increase in the separation distances for the PDMS patterns having a constant pit diameter caused a decrease in most of the contact angles similar to the previously published results on the PDMS patterns made of micropits.^85^

The average apparent θ on the PDMS pattern containing micropits was equal to 127 ± 11°, where θ_a_ = 136 ± 14°, θ_r_ = 89 ± 19°, and CAH = 47 ± 20° as given in Table 4. The deviations from the average θ values were found to be small and around 10% for both apparent θ and θ_a_ values. All the average θ values measured on the PDMS patterns with micro-pits were larger than that of the values obtained on the pillared SU-8 patterns because of the hydrophobicity differences on flat SU-8 (slightly hydrophilic; apparent θ = ∼80° and θ_a_ = ∼90°) and flat PDMS (hydrophobic; apparent θ = 108 ± 1° and θ_a_ = 119 ± 1°). The apparent θ value of 108 ± 1°, which was measured on the flat PDMS, fits the reported value in the literature where apparent θs of 110 ± 2°,^86^ 113.5 ± 2°,^87^ 116.1 ± 0.8°,^88^ and 116 ± 2°^85^ were given on the flat PDMS. θ_a_ = 119 ± 1° and θ_r_ = 95 ± 1° were measured on the flat PDMS as provided in Table 4, similar to the literature reports of θ_a_ = 119 and 120° and θ_r_ = 101° on the flat PDMS.^89^

Moreover, it was found that there were large differences between the water θ results on pillared and pit-type PDMS patterns. Micropit-containing PDMS patterns resulted in 17–26° lower θs than the micropillared PDMS,^90^ and the results in this study were close to the previously published pit-type PDMS pattern data. θ results on the PDMS patterns containing micropit arrays were compared with the literature results given in ref (85), where PDMS micropit arrays with diameters of 20 μm and spacings of 5, 20, and 50 μm were used with a pit depth of 20 μm. θ_a_ = 137 ± 1° was measured for the (dia20-dis20) PDMS sample in this study, whereas θ_a_ = 132 ± 2° was reported in ref (85) for the same-sized pattern. Similarly, θ_r_ = 99 ± 2° was measured in the present study, and θ_r_ = 88 ± 2° was given in ref (85). These values were close to each other, and only a 6° difference was present in the CAH values on the same-sized PDMS patterns.

The large θ_r_ values on the pit-type PDMS patterns indicate the weak pinning of the water droplets on these patterns. The average CAH value obtained on the PDMS micropit patterns was smaller than that of on the pillared SU-8 patterns and had a very high deviation of 43% from the average value as seen for the (dia10-dis20) and (dia15-dis15) samples. This shows the variable pinning behavior of the water droplet on some pit-type patterns depending on the partial filling of the pits with the liquid inside the droplet.^77−79^ Water can penetrate into the pores but not necessarily filling them completely, and some air pockets may be present within the pores depending on pattern uniformity and the conditions during contact angle measurements.^91^

### Pillared PLA Patterns

**Figure 5:**
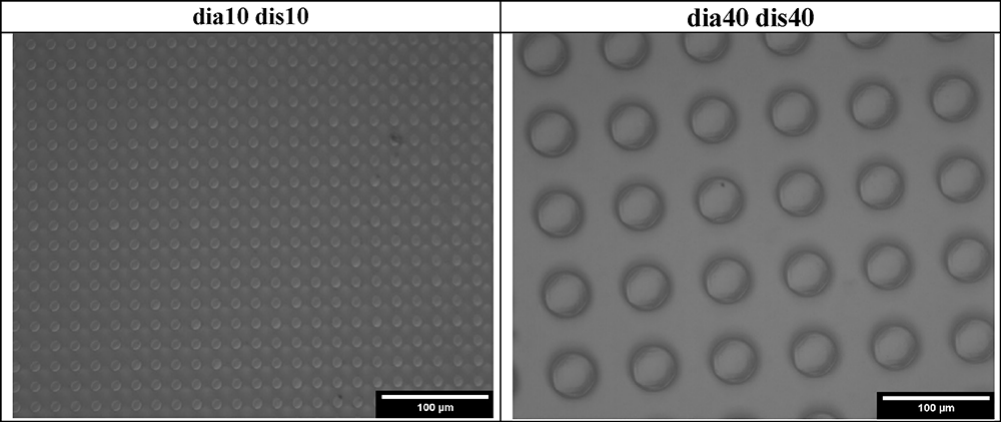
Indicative optical microscope images of two pillared PLA pattern samples.

**Figure 6:**
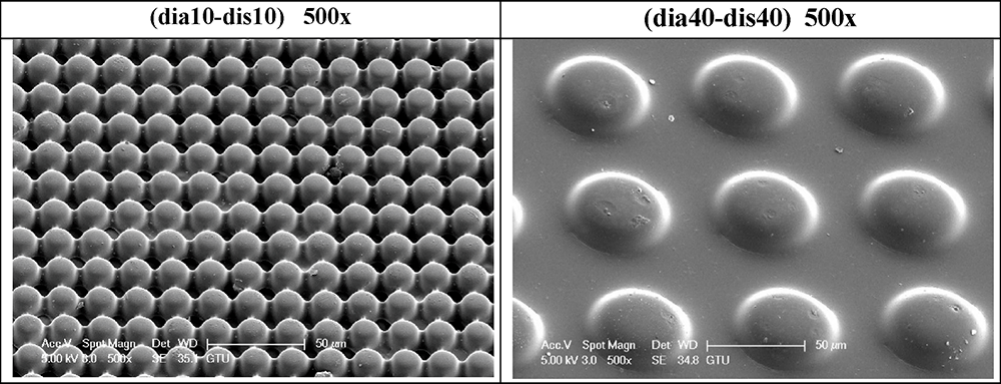
Indicative SEM images of two pillared PLA pattern samples.

Indicative optical microscope images of two pillared PLA pattern samples are given in Figure 5. Optical microscope images of all the pillared PLA pattern samples are given in Figure S9 of the Supplementary Information. Indicative SEM images of two pillared PLA patterns are given in Figure 6. SEM images of the PLA pattern samples of (dia10-dis10) in 250×, 500×, and 1000× magnifications are given in Figure S10, and those of the (dia40-dis40) sample in the same magnifications are given in Figure S11 of the Supporting Information. Uniform cylindrical PLA pillar arrays were seen in all these images with diameters and pillar-to-pillar separation distances fitting the nominal values with an average pillar height of 15 ± 1 μm, indicating that PLA polymer partially filled the pits of the PDMS template.

Indicative images of water droplet profiles on uncoated micropillared PLA patterns are presented in Figure S12 of the Supporting Information. The apparent, advancing, and receding water θ results on the pillared PLA patterns having 15 ± 1 μm height are given in Table 5.

**Table 5:** Apparent, Advancing, and Receding Water θ Results on the Pillared PLA Patterns Having 15 ± 1 μm Height

sample name	apparent θ (°) ± 1	θ_a_ (°) ± 1	θ_r_ (°) ± 2	CAH (°) ± 2
flat PLA	81	93	50	43
dia10-dis10	124	139	95	44
dia10-dis15	127	135	84	51
dia10-dis20	113	122	57	64
dia10-dis25	106	112	47	65
dia15-dis15	117	134	87	47
dia15-dis20	117	119	53	66
dia15-dis25	108	119	53	66
dia20-dis20	120	122	57	66
dia20-dis25	115	121	55	66
dia20-dis30	103	104	54	50
dia25-dis25	117	120	63	57
dia40-dis40	100	103	51	52

**Figure 7:**
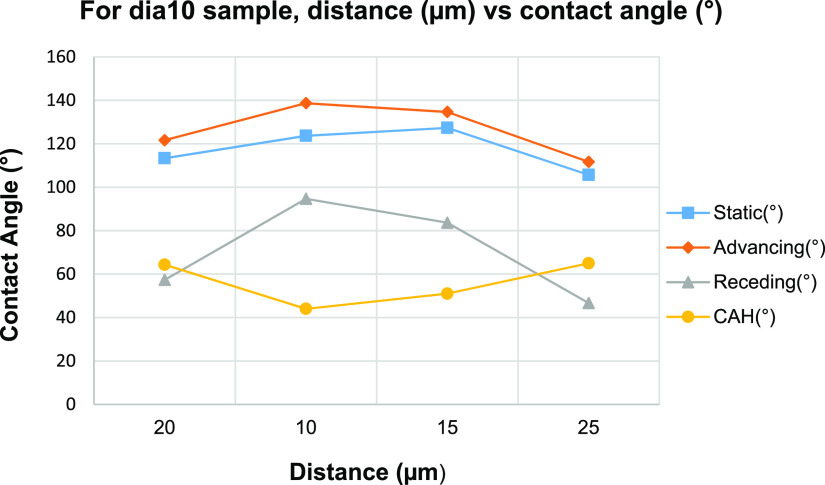
Change of water θ on pillared PLA pattern samples with the change in pillar-to-pillar distance for pillars of 10 μm in diameter.

The results presented in Table 5 indicated that large θ_a_ (134–139°) and θ_r_ (84–95°) values were measured on the PLA patterns made of smaller pillars and shorter pillar-to-pillar distances as seen for (dia10-dis10), (dia10-dis15), and (dia15-dis15) samples. Conversely, small θ_a_ (103 and 104°) and θ_r_ (51–54°) values were measured on the PLA pattern samples made of larger pillars and longer separation distances as seen for (dia25-dis30) and (dia40-dis40) samples. The increase in the pillar-to-pillar distance for the constant pillar diameter of PLA pattern samples resulted in a decrease in all type of the contact angles. When the pillar diameter was kept constant (10 μm), a decrease of the θs on the pillared PLA patterns was seen with the increase of pillar-to-pillar distance as given in Figure 7. It was observed that CAH values increased until the pillar-to pillar distance was 20 μm and then remained almost the same.

The same θ trend was mostly exhibited on other PLA patterns for constant pillar diameter cases. However, the water θ values on the pillared PLA patterns could not be compared with any previously published results due to the lack of data in the existing literature. As seen in Table 5, none of the pillared PLA patterns was superhydrophobic since they had a maximum water θ_a_ of 139°, which was less than the required criteria of 150°.

The average apparent θ on the pillared PLA patterns was equal to 114 ± 13°, where θ_a_ = 121 ± 18°, θ_r_ = 63 ± 24°, and CAH = 58 ± 11° as given in Table 5. The deviations from the average θ values were found to be 11% for the apparent θ and 15% for θ_a_. It was also determined that the average θ values measured on the pillared PLA patterns were approximately 10° smaller than that of the θ values obtained for the pillared SU-8 patterns (except CAH values), although both apparent θ and θ_a_ values on the flat PLA and SU-8 samples were very close to each other. Thus, this 10° difference in θs cannot be related to the chemistry of the polymeric pillars but to their heights and the geometric forms of the top of the pillars. PLA pillars had a slightly curved shape at the top, resembling a flattened hemisphere, and SU-8 had a cylindrical shape at the top, which would increase the drop pinning and result in higher apparent θ and θ_a_.

The apparent θ on the flattened PLA layer was measured to be 81 ± 1°, which fits the value reported in the literature for the apparent θ on a flat PLA as 80 ± 1°.^92^ In the present study, θ_a_ and θ_r_ values on the flattened PLA were measured to be 93 ± 1 and 50 ± 2°, respectively, as seen in Table 5, which were very close to the reported values of 91 and 50° in the literature.^93^ θ values on the flat PLA were used to calculate the theoretical contact angles on the pillared PLA patterns using Cassie–Baxter and Wenzel equations and are given in Table S3 of the Supporting Information. None of the experimental apparent θ results on the pillared PLA patterns fit the theoretical θ estimates as expected.^22,26,77−81^ The large deviation of apparent contact angles on the pillared PLA patterns from the Cassie–Baxter equation is given in Figure S13.

### DMDCS, NPTS, and TDFS Silane-Coated SH-PLA Patterns Containing Pillars

**Figure 8:**
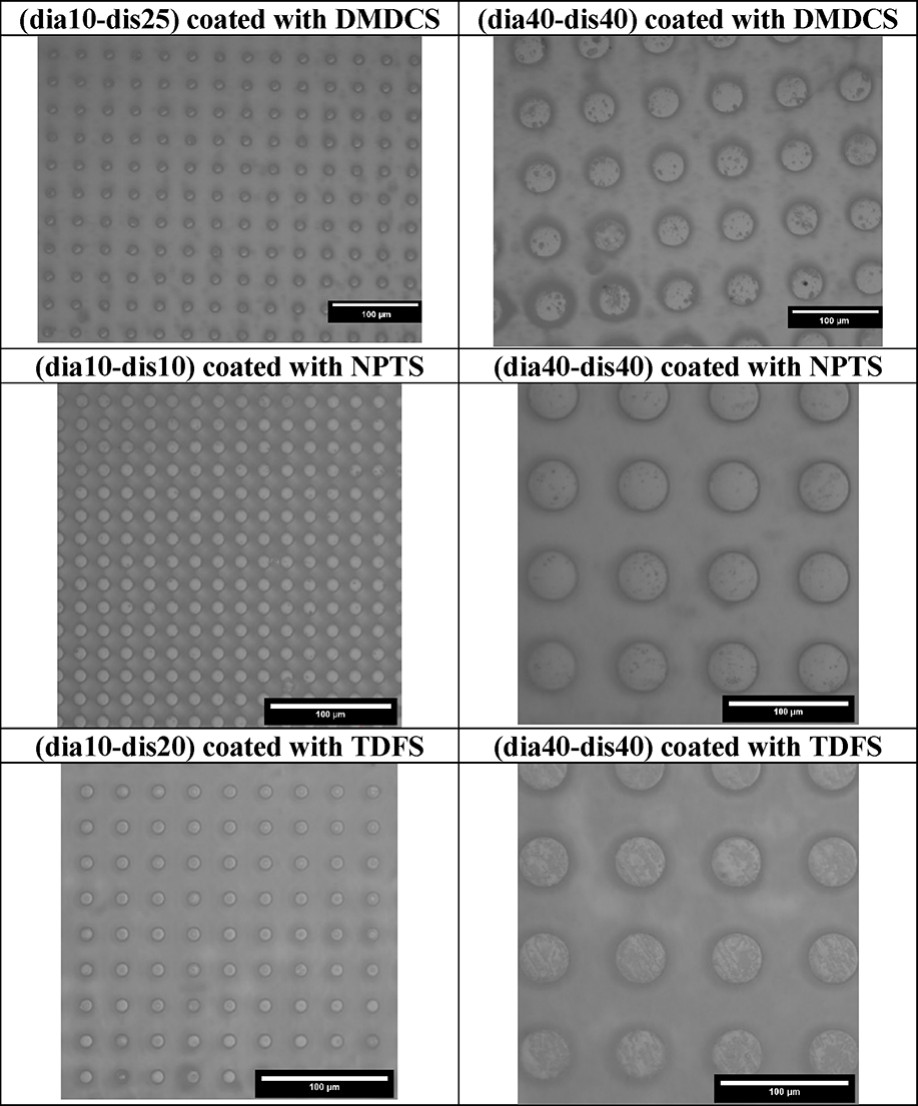
Indicative optical microscope images of the pillared PLA patterns coated with silanes.

Since no SH-PLA pattern having a water θ larger than 150° could be obtained by using the pure PLA polymer in pillared pattern preparation giving a maximum water θ_a_ of 139°, the produced PLA pattern samples were coated by three different silanes by applying the CVD method to increase their hydrophobicities. Indicative optical microscopy images of six silane-coated pillar-type PLA patterns are given in Figure 8.

**Figure 9:**
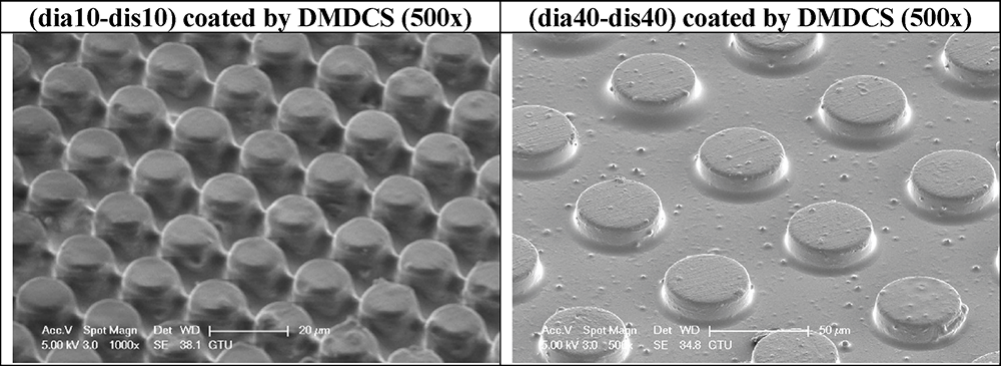
Indicative SEM images of two pillared PLA patterns coated with DMDCS.

**Figure 10:**
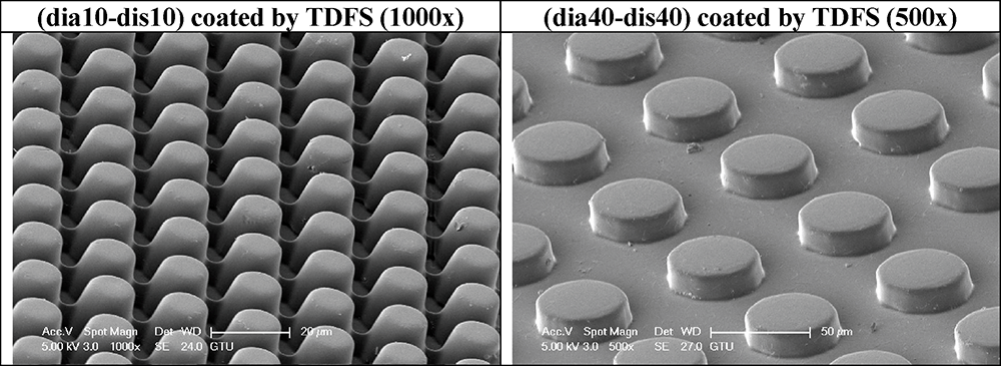
Indicative SEM images of two pillared PLA patterns coated with TDFS.

The optical microscopy images of the DMDCS-, NPTS-, and TDFS-coated pillared PLA patterns were presented in Figures S14, S15, and S16 of the Supplementary Information, respectively. Indicative SEM images of the DMDCS-coated pillar-type PLA pattern samples of (dia10-dis10) and (dia40-dis40) are given in Figure 9, and those of the TDFS-coated pattern samples in Figure 10. Other SEM images of the PLA pattern samples (dia10-dis10) and (dia40-dis40) coated with DMDCS at 250×, 500×, and 1000× magnification are given in Figures S17 and S18 of the Supporting Information, respectively.

In addition, SEM images of the TDFS-coated PLA pattern samples (dia10-dis10) and (dia40-dis40) at 250×, 500×, and 1000× magnification are exhibited in Figures S19 and S20 of the Supporting Information, respectively.

As seen in the images given in Figure 9 and Figures S14 and S15, the thickness of the DMDCS coating was higher than that of the TDFS coating because of the heavy deposition of the DMDCS silane layer on the PLA patterns during the CVD process as given in Table S1 of the Supporting Information. The presence of many triangular DMDCS protrusions (probably DMDCS agglomerates) on the PLA surfaces was seen, which generates pillars with rough tops. However, nearly no TDFS agglomerate was seen on the coated PLA patterns in the images given in Figure 10 and Figures S19 and S20, indicating a thin and uniform TDFS coating on the pillars.

**Figure 11:**
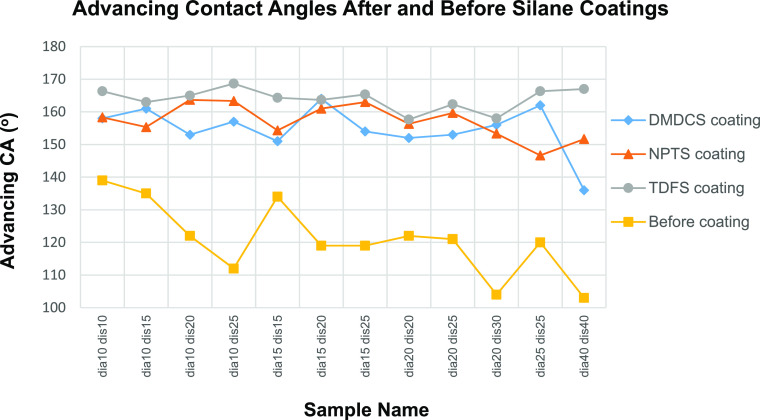
Comparison of θ_a_ values on the pillared PLA patterns after coating with three silanes using the CVD method.

Indicative images of water droplet profiles on the micropillared PLA patterns after coating with DMDCS, NPTS, and TDFS are given in Figure S21 of the Supporting Information. The advancing and receding water θs, which were measured on the pillared PLA pattern samples after they were coated by three different silanes, are given in Table 6. The θ_a_ results of each coating are compared in Figure 11.

**Table 6:** Advancing and Receding Contact Angle Values of Pillared PLA Pattern Samples after the Coating with Three Different Silanes

	DMDCS		NPTS		TDFS	
sample name	θ_a_ (°) ± 1	θ_r_ (°) ± 2	θ_a_ (°) ± 1	θ_r_ (°) ± 2	θ_a_ (°) ± 1	θ_r_ (°) ± 2
flat silane-coated PLA	110	87	107	74	124	100
dia10-dis10	158	110	158	142	166	139
dia10-dis15	161	99	155	145	163	141
dia10-dis20	153	93	164	102	165	134
dia10-dis25	157	90	163	136	169	136
dia15-dis15	151	107	154	133	164	137
dia15-dis20	164	108	161	135	164	139
dia15-dis25	154	82	163	97	165	143
dia20-dis20	152	122	156	132	158	146
dia20-dis25	153	103	160	143	162	137
dia20-dis30	156	84	153	91	158	134
dia25-dis25	162	104	147	105	166	146
dia40-dis40	136	78	152	99	167	136

The highest θ_a_ values on the pillared PLA patterns were obtained by coating with TDFS, which has water-repellent fluorine atom in its structure. The lowest θ_a_ results were measured with DMDCS coating as seen in Figure 10. The apparent water contact angle and CAH values, which were determined on the coated PLA pillar type pattern samples by three different silanes, are given in Table 7.

**Table 7:** Apparent Contact Angle and CAH Values of Patterned PLA Samples after Coating with Three Different Silanes

	DMDCS		NPTS		TDFS	
sample name	apparent θ (°) ± 1	CAH (°) ± 2	apparent θ (°) ± 1	CAH (°) ± 2	apparent θ (°) ± 1	CAH (°) ± 2
flat silane-coated PLA	105	23	102	33	113	24
dia10-dis10	150	48	154	16	154	27
dia10-dis15	151	62	152	10	151	22
dia10-dis20	150	60	151	63	156	31
dia10-dis25	154	67	151	28	160	32
dia15-dis15	153	42	153	21	155	27
dia15-dis20	154	56	151	26	154	25
dia15-dis25	152	72	155	66	152	22
dia20-dis20	152	30	154	25	153	12
dia20-dis25	152	50	153	16	152	26
dia20-dis30	153	72	151	62	153	24
dia25-dis25	154	58	153	42	151	20
dia40-dis40	132	57	151	52	151	31

In summary, superhydrophobic PLA pattern surfaces were obtained for all the silane-coated samples except the (dia40-dis40) sample coated with DMDCS and the (dia25-dis25) sample coated with NPTS as seen in Tables 6 and 7. The apparent θ on the flat PLA layer coated with DMDCS was measured to be 105 ± 1° as given in Table 7, which fits the value reported in the literature for the apparent θ on a DMDCS-coated flat Si-wafer surface as 106 ± 1°.^78^ In the present study, the θ_a_ and θ_r_ values on the DMDCS-coated PLA layer were measured to be 110 ± 1 and 87 ± 2°, respectively, as seen in Table 6, where the θ_a_ value is close to the reported value of 106 ± 1°. However, the θ_r_ value was much lower than the value of 102 ± 2° given in the literature.^78^ The reason of this difference may be the presence of DMDCS protrusions on our PLA patterns, which caused a strong pinning effect and decreased the θ_r_ value. For the NPTS-coated flat PLA layers, the apparent θ = 102 ± 1° (Table 7) was measured, which was 5° larger than the apparent θ value reported in the literature on a NPTS-coated flat Si-wafer surface as 97 ± 1°.^94^ We also found θ_a_ = 107 ± 1° and θ_r_ = 74 ± 2° values on a NPTS-coated flat PLA layer (Table 6). However, no data is available to compare in the literature. For the TDFS-coated flat PLA layers, the apparent θ = 113 ± 1° (Table 7), which was very close to the value reported in the literature for the apparent θ on a TDFS-coated glass surface, θ > 110°.^95^ We also measured θ_a_ = 124 ± 1° and θ_r_ = 100 ± 2° values on a TDFS-coated PLA surface (Table 6). The higher values of θ of the TDFS-coated flat samples were attributed to the presence of highly water-repellent fluorine atoms in the TDFS structure.

As seen in Tables 6 and 7, the average apparent θ on the DMDCS-coated PLA patterns was equal to 151 ± 11°, where θ_a_ = 155 ± 14°, θ_r_ = 98 ± 22°, and CAH = 56 ± 21°. The deviations from the average values were found to be low: 7% for the apparent θ and 9% for θ_a_. The average apparent θ on the NPTS-coated PLA patterns was equal to 152 ± 2°, where θ_a_ = 157 ± 8°, θ_r_ = 122 ± 27°, and CAH = 36 ± 28°. The deviations from the average values were found to be considerably low: 1.3% for the apparent θ and 5% for θ_a_. The average apparent θ on the TDFS-coated PLA patterns was equal to 154 ± 4°, where θ_a_ = 164 ± 5°, θ_r_ = 139 ± 6°, and CAH = 25 ± 10°. The deviations from the average values were found to be very low: 2.5% for the apparent θ and 3% for θ_a_. Since protrusions of silane agglomerates were formed during the DMDCS coating onto PLA, the deviations of all of the θ values from the average were large for this type of silane coating. In comparison, the NPTS coating increased slightly both average apparent θ and θ_a_ values than that of the DMDCS-coated PLA patterns. However, the increase of average was large (around 24°) and thus resulted in a considerable decrease of CAH values (around 20°). On the other hand, the highest apparent θ, θ_a_, and θ_r_ values were obtained on the TDFS-coated pillared PLA patterns with the corresponding lowest CAH values. This was expected since TDFS contains water-repellent fluorine atoms in its structure. But the water θ values on the silane-coated PLA pillar type patterns could not be compared with previously published results due to the lack of data in the existing literature.

It was determined that the average apparent θ and θ_a_ values measured on the silane-coated PLA pillar-type patterns are 30–40° larger than that of the values obtained on the pillared SU-8 and uncoated PLA patterns. This was expected since the silane coatings increased the hydrophobicity of the pillars considerably. However, attention should also be paid to the height differences of the pillars. PLA pillars were shorter (∼15 μm) than the SU-8 pillars (∼21 μm) and this is a factor to decrease the water θ values. The high contact angles of 164–167°, which were obtained on the silane-coated PLA pillars, indicate the success of achieving SH surfaces by the application of a simple CVD silane coating. Washing stability tests were also carried out for three silane coatings on both flat and pillared PLA patterns by applying initial ethanol washing following pressurized pure water washing tests, and only 1–2° of contact angle decrease was found after the tests. We also evaluated the loss of the coating under ambient conditions (service life of the coated PLA patterns) and determined that there was no water θ decrease for all silane-coated PLA pattern samples after 6 months.

Apparent θs that were obtained on the flat silane-coated PLA layers were used to calculate the theoretical Cassie–Baxter and Wenzel θs on the pillared silane-coated PLA patterns. The results are given in comparison with the experimental values in Tables S4–S6 and Figures S18–S20 of the Supporting Information. It was found that none of the experimental θ results on the silane-coated PLA patterns fit the theoretical θ from the Wenzel equation as expected. Only few results agreed with the Cassie–Baxter equation as seen in the deviations in Figures S22–S24 through the blue dotted line representing the theoretical Cassie–Baxter equation.

## Conclusions

In this study, the pillared PLA patterns were prepared by hot-pressing pre-flattened PLA sheets onto the preformed PDMS templates having micropits with 12 different diameters and pit-to-pit distances. Apparent θ, θ_a_, and θ_r_ measurements of water droplets were made on the PLA patterns, and optical microscopy and SEM were applied to examine the morphology of the patterns. No SH-PLA pattern sample could be obtained without the surface modification of the uncoated PLA patterns (largest θ_a_ < 139°). For this reason, surface hydrophobizations using three different silanes were applied to obtain the SH-PLA patterns having θ_a_ > 150°. The highest contact angle values were obtained (θ_a_ = 167°) with the corresponding lowest CAH values when the PLA pattern samples were coated with TDFS having a fluorine atom in its chemical structure. NPTS coatings were found to be uniform, robust, and environmentally friendly. DMDCS coatings on PLA resulted in agglomerated thick coatings, giving a two-ranged surface morphology that was not appropriate since the deviations of all of the θ values from the average were large for DMDCS-coated PLA pattern samples. Washing and service life stability tests were also applied to the coated samples. It was determined that all silane coatings on PLA were not removed from the PLA surface for 6 months.

## Supporting Information Available

Indicative view of chrome mask design; image of micropatterned PLA sample; optical microscope images of SU-8 micropillars, PDMS micropits, uncoated PLA, and DMDCS-, NPTS-, and TDFS-coated PLA by using the CVD method; water droplet profiles used in contact angle measurements; SEM images of the PDMS mold, uncoated PLA, and DMDCS-, NPTS-, and TDFS-coated PLA samples; tables and graphics of the experimental contact angle data comparison with Wenzel and Cassie–Baxter theoretical angles (PDF)
