# Supplementary material for: Wetting of Superhydrophobic Polylactic Acid Micropillared Patterns
Source: Langmuir. 2022 Aug 5;38(32):10052–64. doi: 10.1021/acs.langmuir.2c01708 (PMC9387099; doi:10.1021/acs.langmuir.2c01708)
Supplement: Supplementary file 1 — la2c01708_si_001.pdf [file la2c01708_si_001.pdf]

SUPPORTING INFORMATION

Wetting of Superhydrophobic Polylactic Acid (PLA) Micropillared Patterns

Eda Hazal Tümer[a], H. Yildirim Erbil[a*], Numan Akdoğan[b]

[a] Department of Chemical Engineering, Gebze Technical University, 41400 Gebze, Kocaeli, Türkiye

[b] Department of Physics, Gebze Technical University, 41400 Gebze, Kocaeli, Türkiye

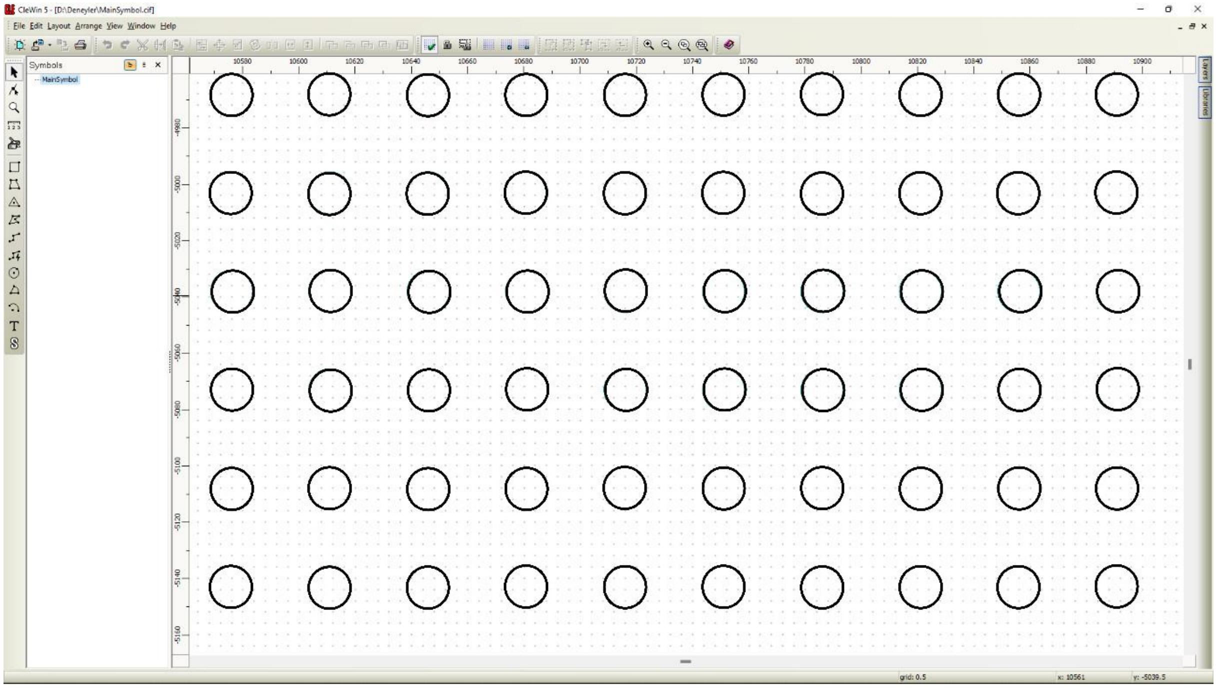

Figure S1. An indicative view of chrome mask design for SU-8 patterns

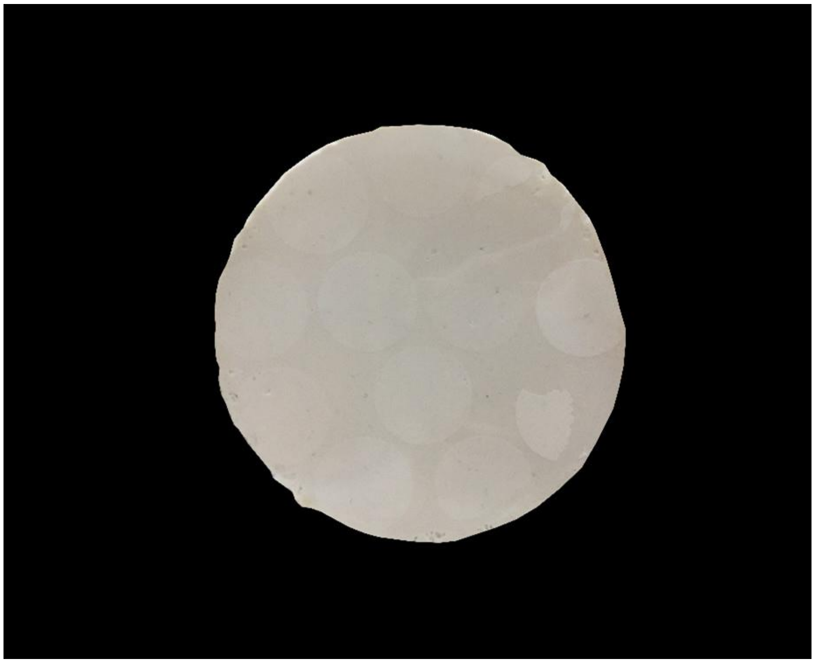

Figure S2. PLA sample after micro-patterning by thermo-pressing onto the PDMS template.

Sample Name	200x	Sample Name	200x
**dia10-dis10**	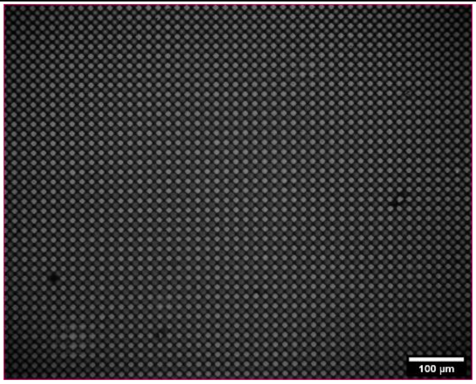	**dia10-dis15**	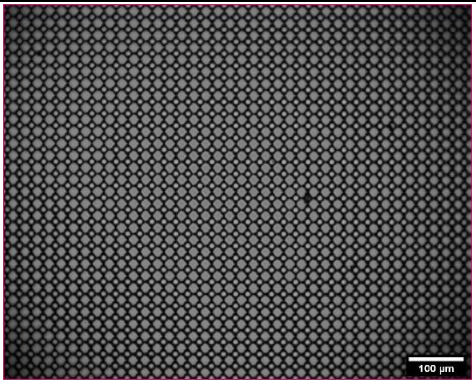
**dia10-dis20**	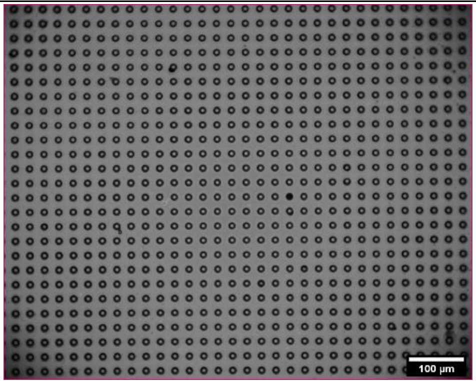	**dia10-dis25**	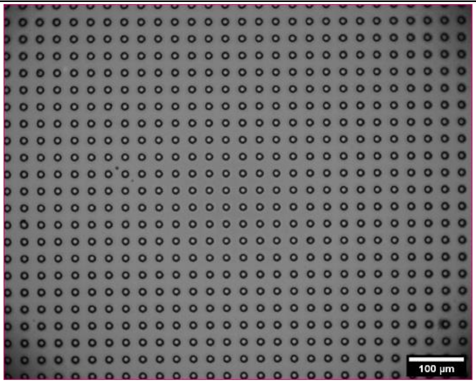
**dia15-dis15**	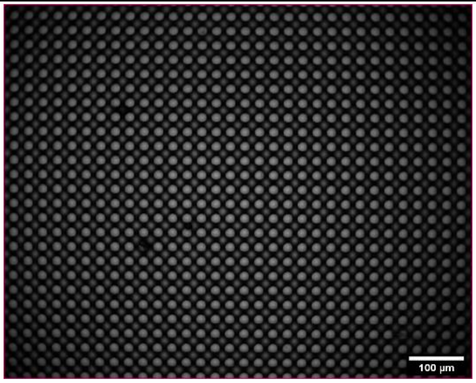	**dia15-dis20**	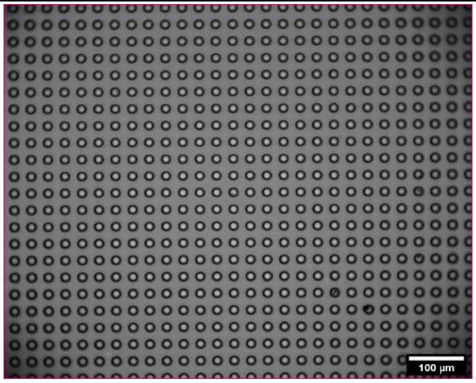
**dia15-dis25**	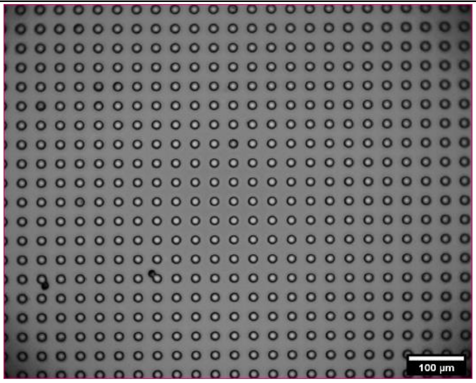	**dia20-dis20**	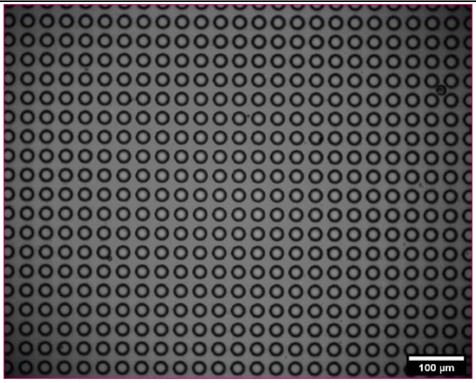

**dia20-dis25**	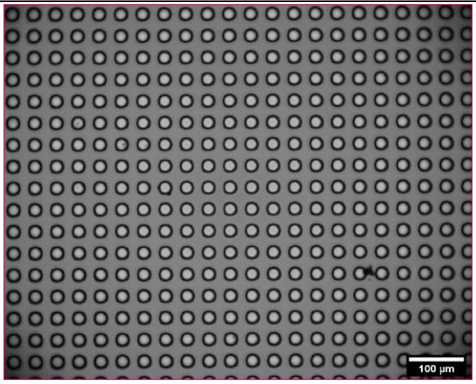	**dia20-dis30**	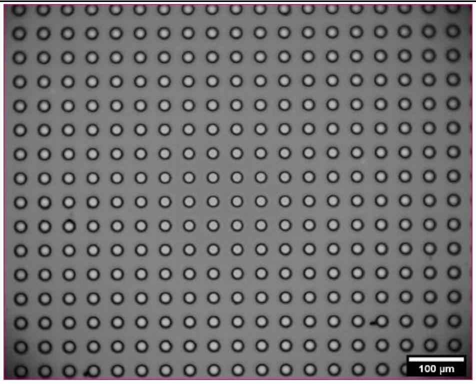
**dia25-dis25**	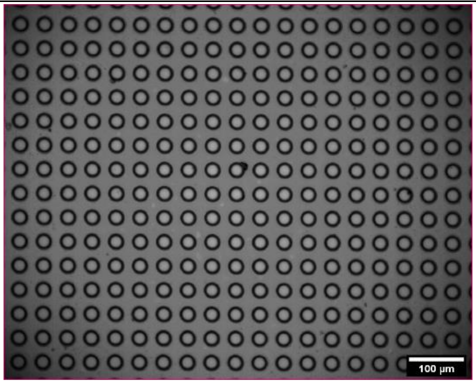	**dia40-dis40**	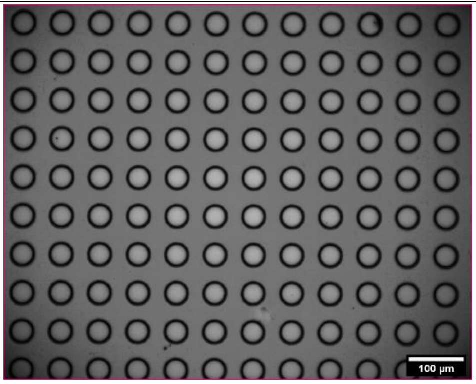

Figure S3. Optical microscope images of the pillared SU-8 patterns.

dia10-dis15	dia40-dis40
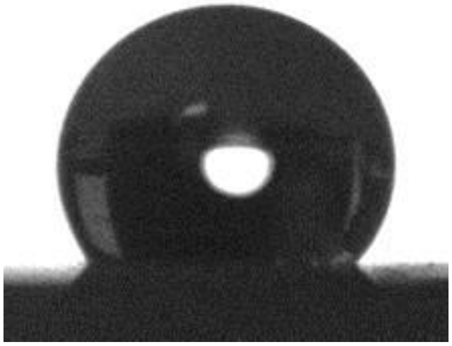	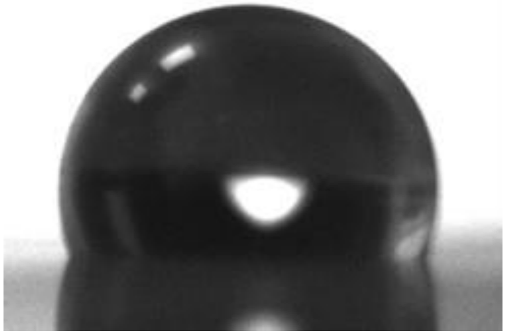

Figure S4. Indicative images of water droplet profiles on the SU-8 patterns.

Table S1: Boiling points of silanes used in CVD application with deposition mass (%).

Silane	Silane Name	Boiling Point	Silane deposited on PLA surface after CVD (mass %)
DMDCS	Dimethyldichlorosilane	70ºC	35.0
TDFS	(Tridecafluoro-1,1,2,2- tetrahydrooc-tyl)trichlorosilane	84-85°C (17mmHg)	2.2
NPTS	n-propyltrichlorosilane	123°C	2.1

Table S2. Theoretical contact angles which were calculated by using Cassie-Baxter and Wenzel equations on the pillared SU-8 patterns in comparison with the experimental contact angle values.

Sample Name	Cassie θ (°)	Wenzel θ (°)	Measured θ_{app} (°) ± 1	Cassie-% Deviation	Wenzel-% Deviation
dia10-dis10	140	63	137	2,2	54,0
dia10-dis15	149	69	131	13,3	47,3
dia10-dis20	154	72	122	26,1	40,6
dia10-dis25	158	75	115	37,1	35,2
dia15-dis15	140	69	126	11,4	45,5
dia15-dis20	146	72	128	14,2	44,0
dia15-dis25	151	74	111	35,6	33,6
dia20-dis20	140	72	134	4,7	46,6
dia20-dis25	145	73	124	16,8	40,9
dia20-dis30	145	75	114	30,3	34,5
dia25-dis25	140	73	120	16,9	39,0
dia40-dis40	140	76	112	25,0	32,2

**Sample Name**	**200x**	**Sample Name**	**200x**
**dia10-dis10**	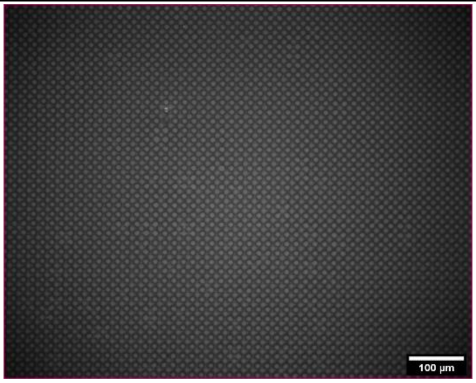	**dia10-dis15**	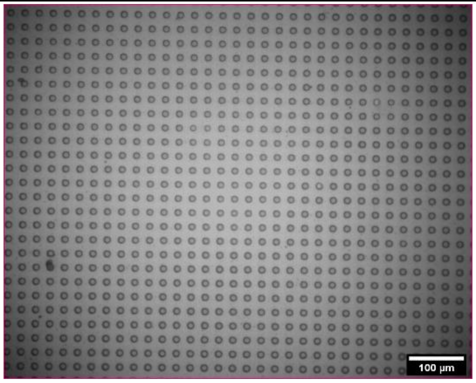
**dia10-dis20**	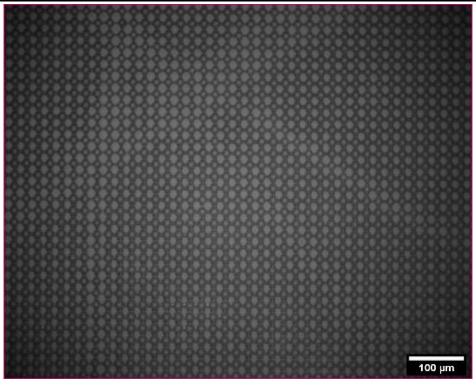	**dia10-dis25**	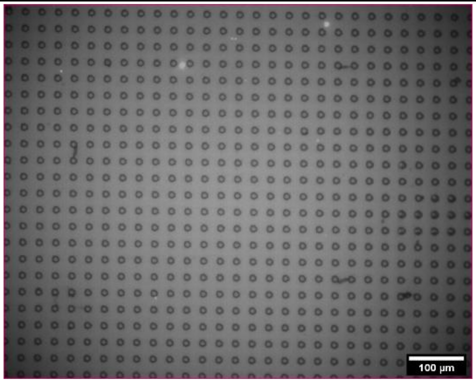
**dia15-dis15**	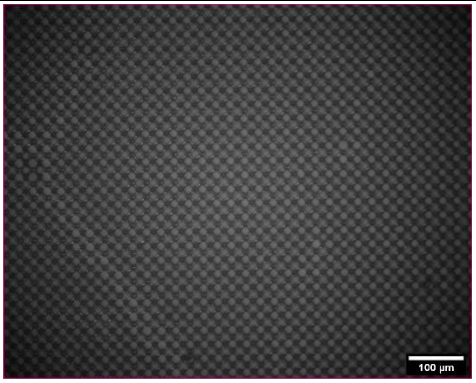	**dia15-dis20**	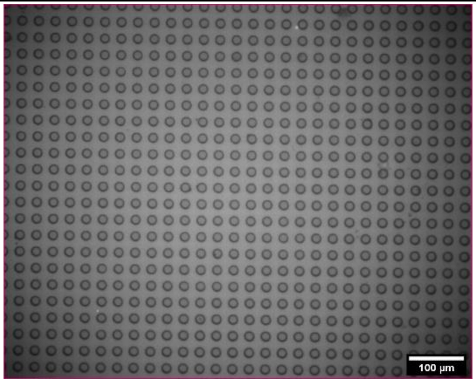
**dia15-dis25**	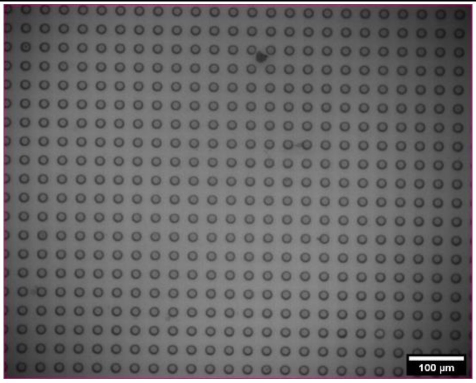	**dia20-dis20**	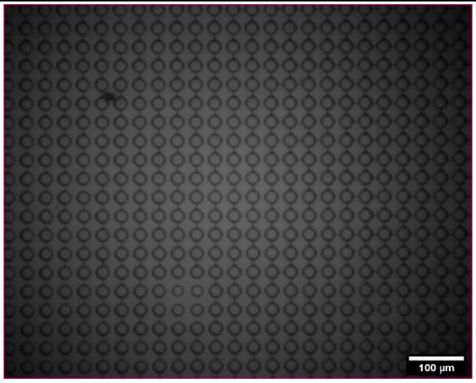

**dia20-dis25**	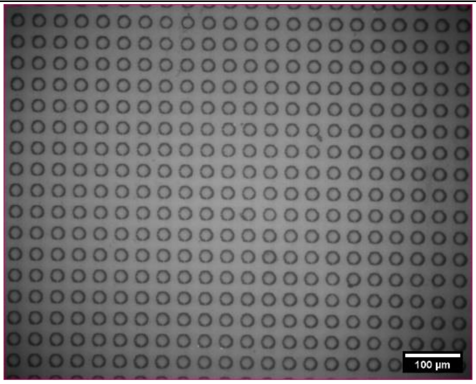	**dia20-dis30**	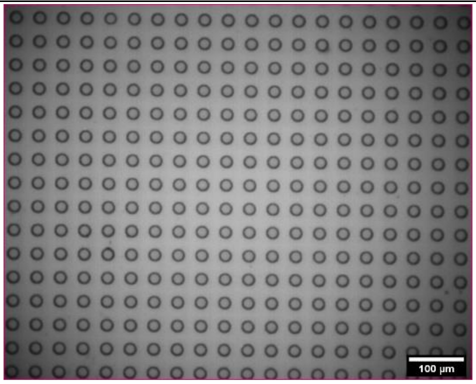
**dia25-dis25**	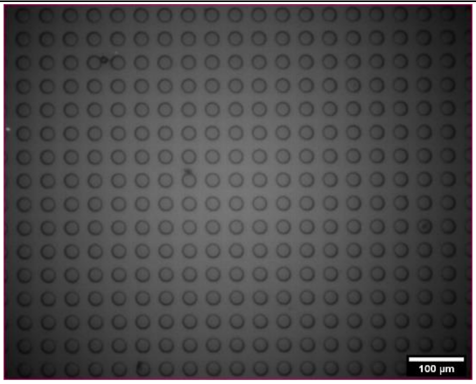	**dia40-dis40**	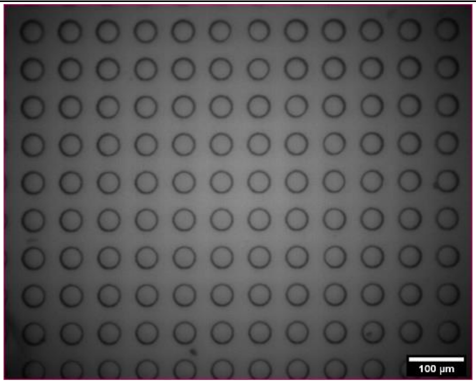

Figure S5. Optical microscope images of the PDMS patterns containing micro-pits.

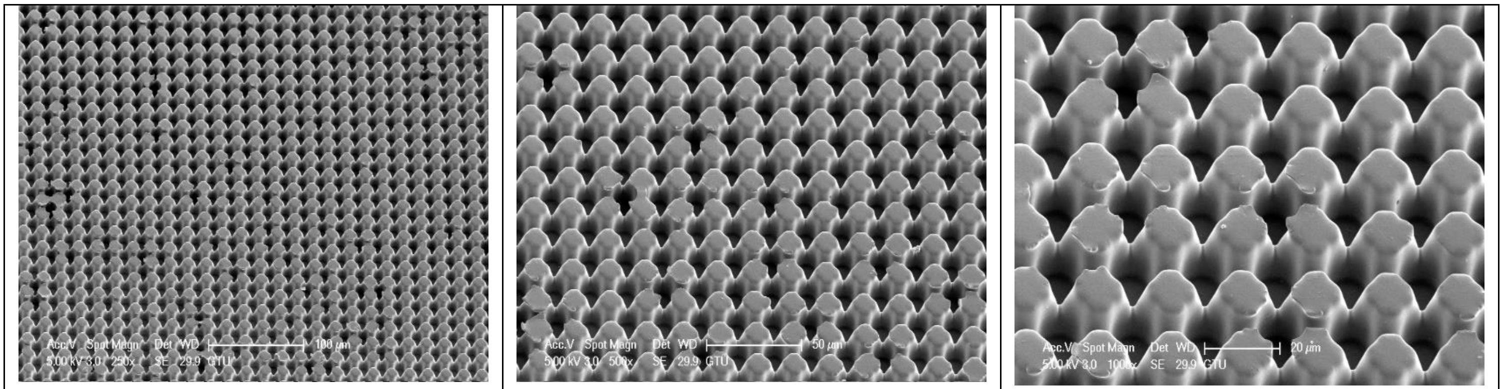

Figure S6. SEM images of the PDMS template with pit diameter=10 µm and pit-to-pit distance=10 µm for 250x, 500x and 1000x magnifications.

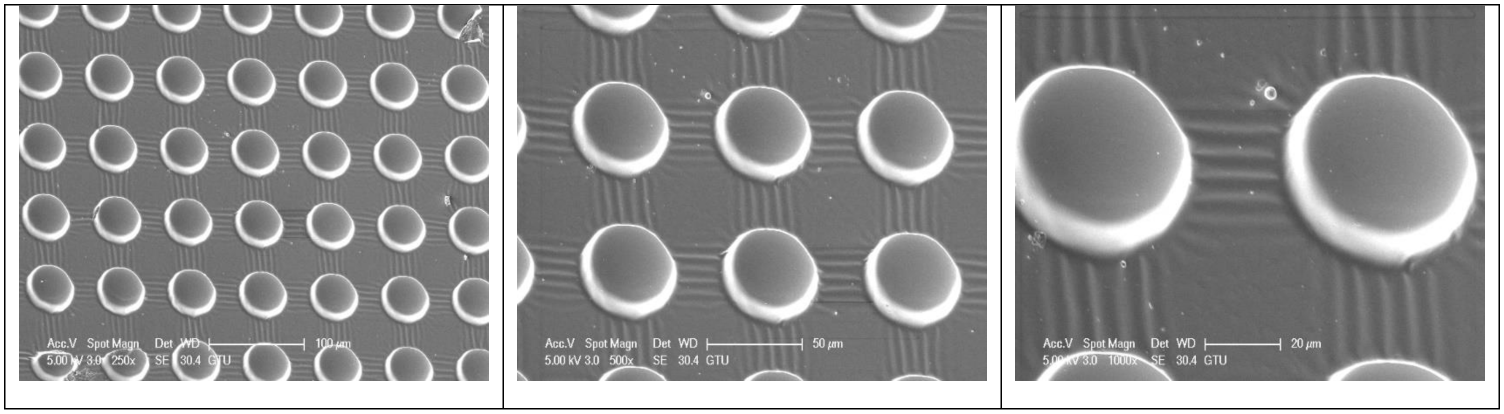

Figure S7. SEM images of PDMS template with pit diameter=40 µm, and pit-to-pit distance=40 µm for 250x, 500x and 1000x magnifications.

dia10-dis10	dia40-dis40
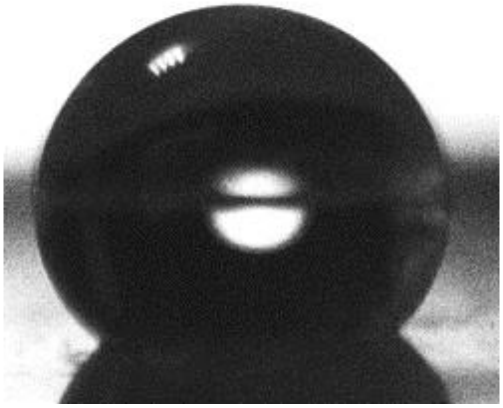	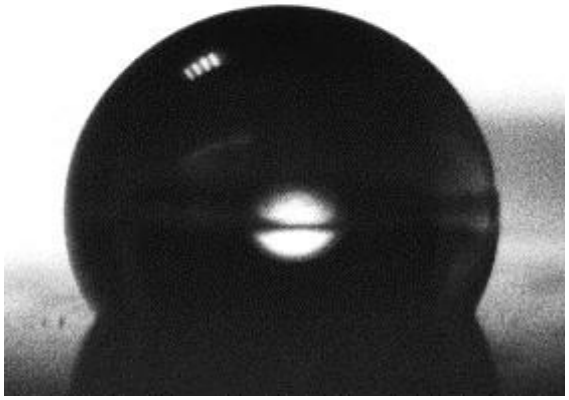

Figure S8. Indicative images of water droplet profiles on the PDMS micro-pit patterns.

**Sample Name**	**200x**	**Sample Name**	**200x**
**dia10-dis10**	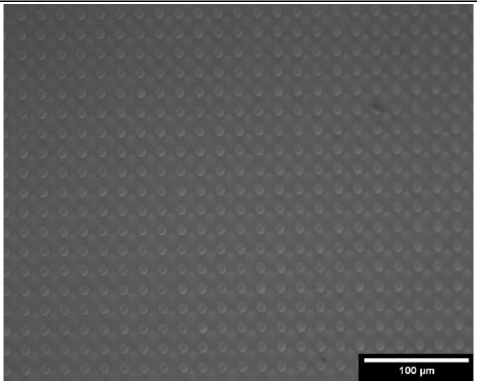	**dia10-dis15**	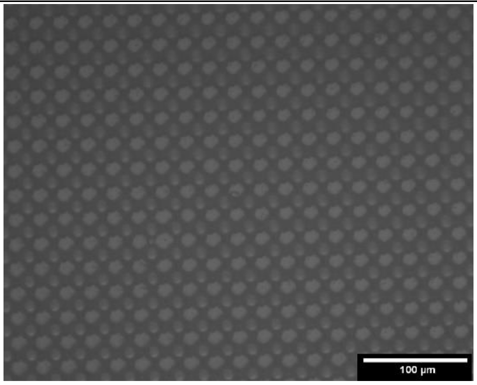
**dia10-dis20**	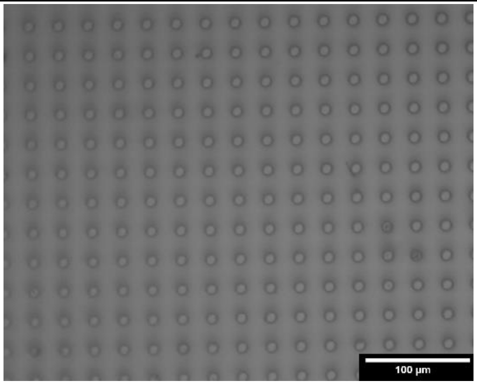	**dia10-dis25**	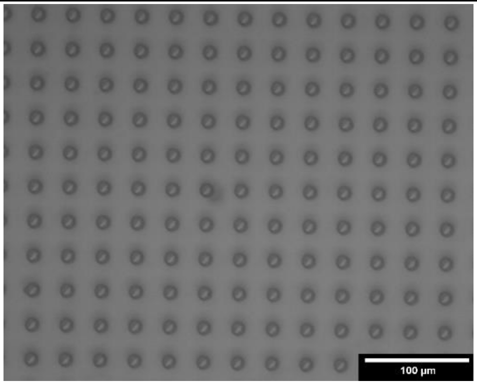
**dia15-dis15**	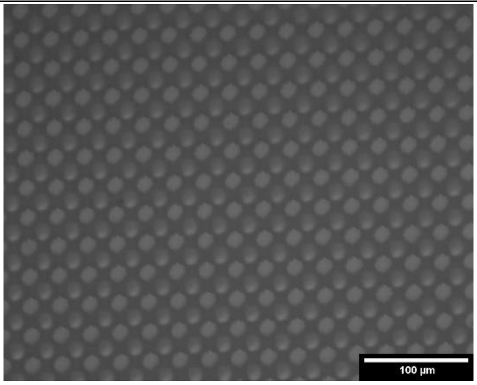	**dia15-dis20**	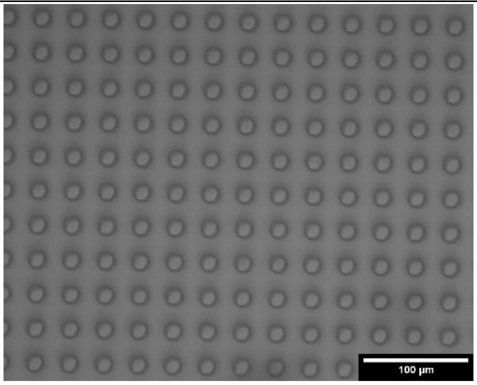
**dia15-dis25**	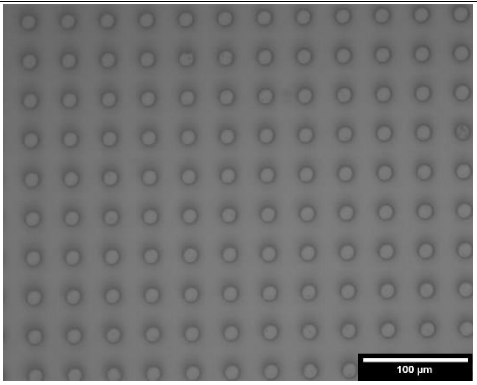	**dia20-dis20**	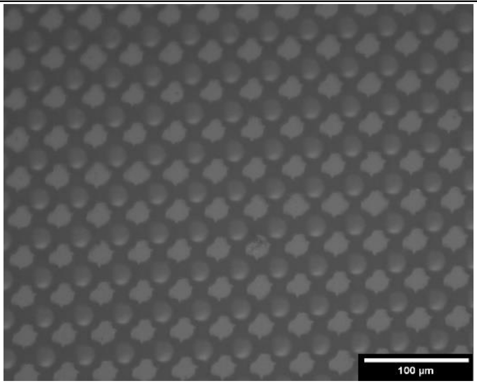

**dia20-dis25**	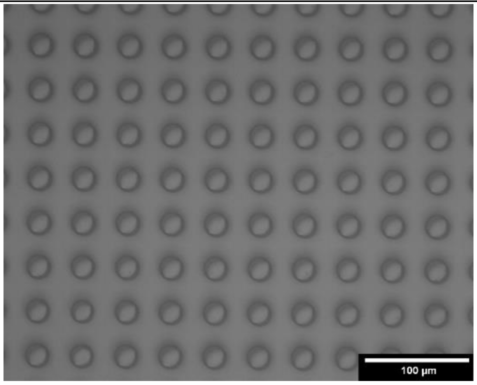	**dia20-dis30**	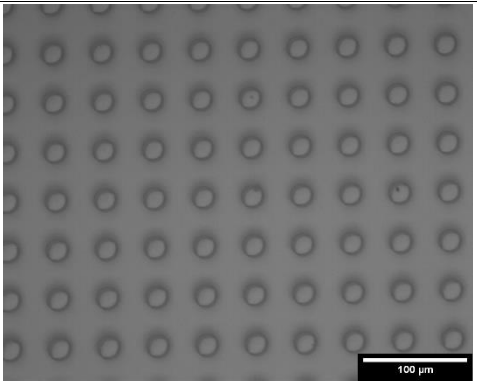
**dia25-dis25**	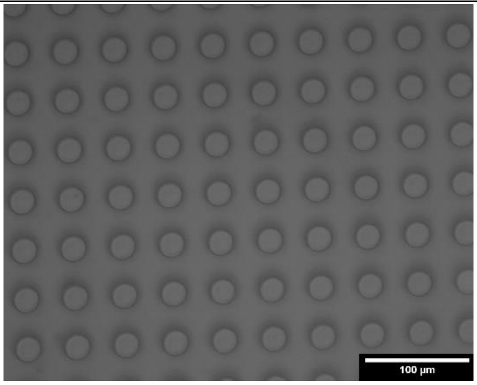	**dia40-dis40**	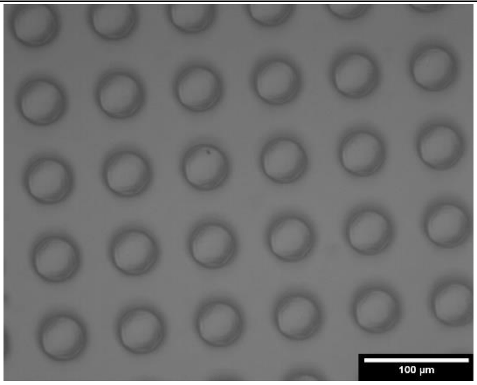

Figure S9. Optical microscope images of pillared PLA patterns.

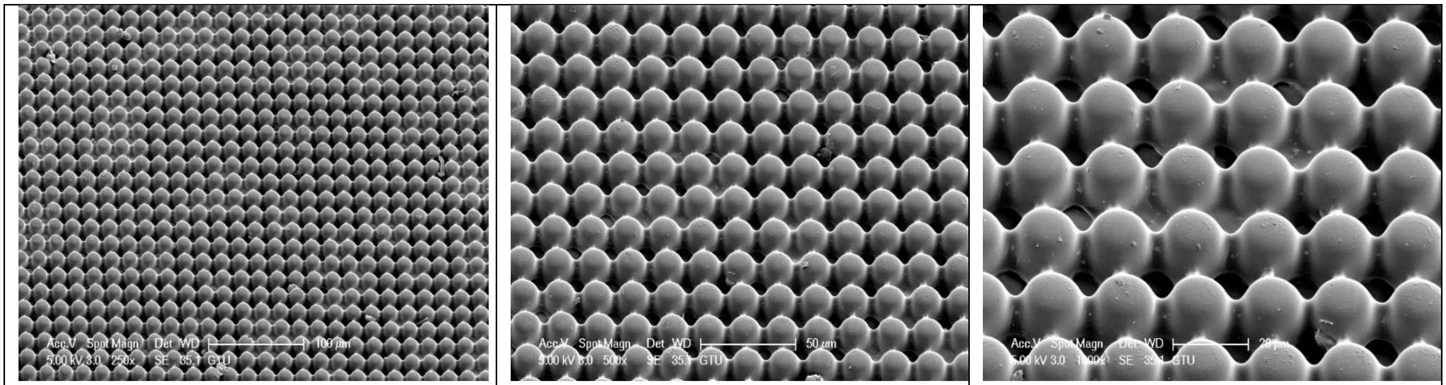

Figure S10. SEM images of uncoated PLA pattern with pillar diameter=10 µm and pillar-to-pillar distance=10 µm for 250, 500 and 1000x magnifications.

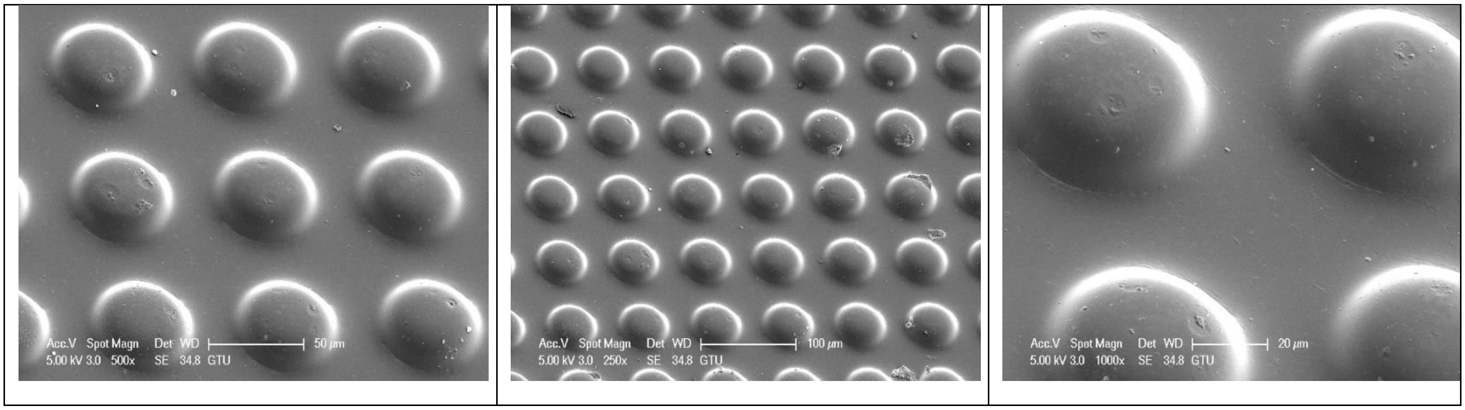

Figure S11. SEM images of uncoated PLA pattern with pillar diameter=40 µm, and pillar-to-pillar distance=40 µm for 250, 500 and 1000x magnifications.

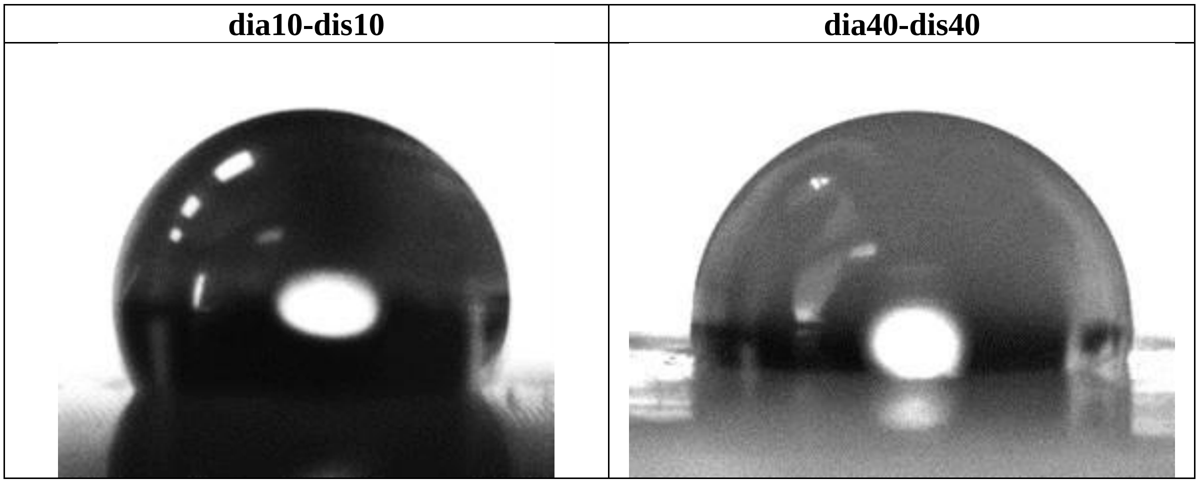

Figure S12. Indicative images of water droplet profiles on uncoated micropillared PLA patterns.

Table S3. Theoretical contact angles which were calculated by using Cassie-Baxter and Wenzel equations on the pillared PLA patterns in comparison with the experimental values.

Sample Name	Cassie θ (°)	Wenzel θ (°)	Measured θ_{app} (°) ± 1	Cassie-% Deviation	Wenzel-% Deviation
dia10-dis10	141	70	124	13,7	43,5
dia10-dis15	149	74	127	17,1	41,7
dia10-dis20	154	76	113	36,3	32,6
dia10-dis25	158	77	106	48,9	26,9
dia15-dis15	141	74	117	20,2	36,9
dia15-dis20	146	76	117	25,2	35,3
dia15-dis25	151	77	108	39,6	28,7
dia20-dis20	141	76	120	17,2	37,0
dia20-dis25	145	77	115	26,2	33,3
dia20-dis30	149	78	103	44,3	24,7
dia25-dis25	141	77	117	20,2	34,4
dia40-dis40	141	78	100	41,0	22,0

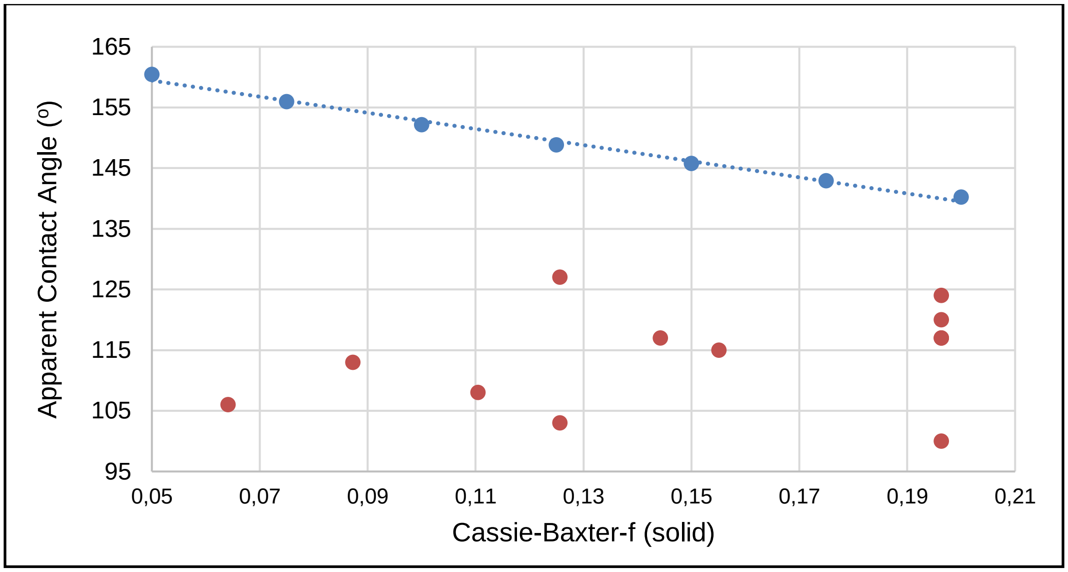

Figure S13. The deviation of apparent contact angles on the pillared PLA patterns from the Cassie-Baxter equation (blue dotted line).

**Sample Name**	**200x**	**Sample Name**	**200x**
**dia10-dis10**	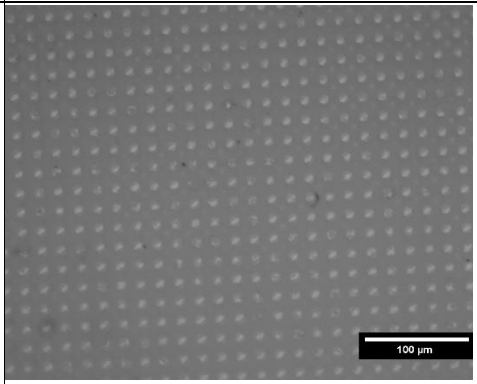	**dia10-dis15**	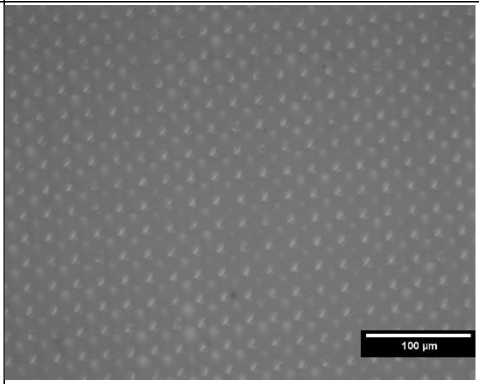
**dia10-dis20**	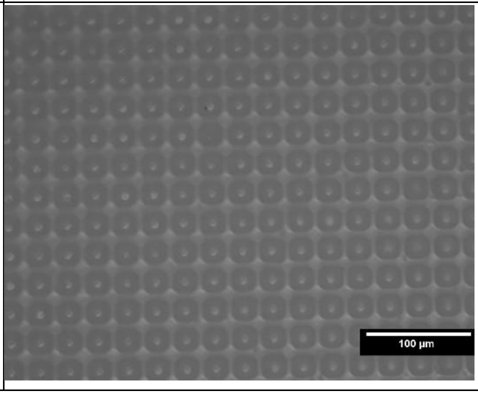	**dia10-dis25**	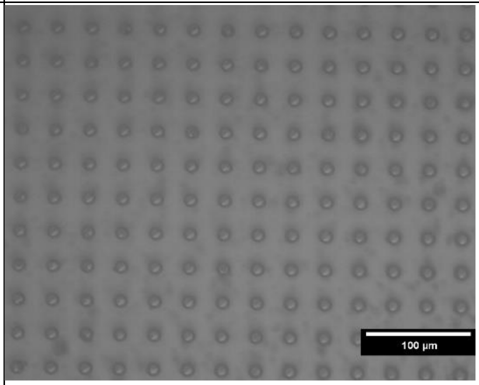

**dia15-dis15**	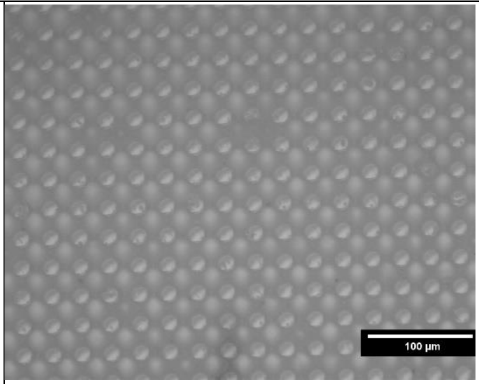	**dia15-dis20**	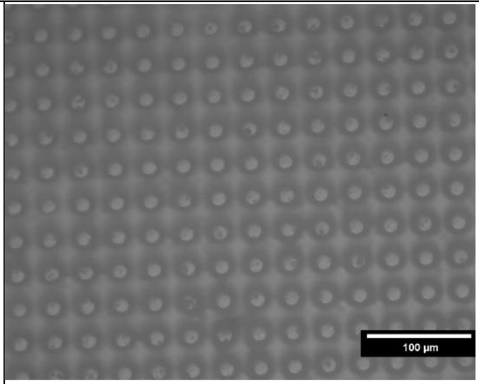
**dia15-dis25**	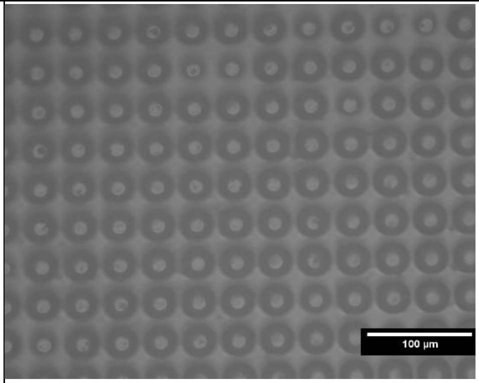	**dia20-dis20**	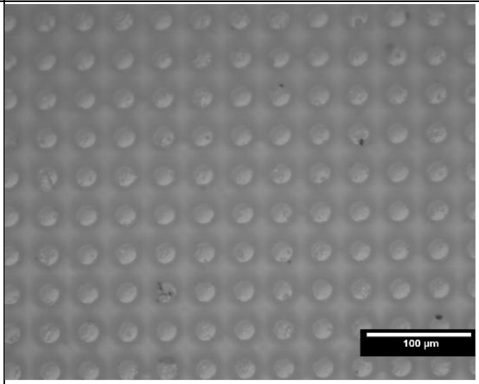
**dia20-dis25**	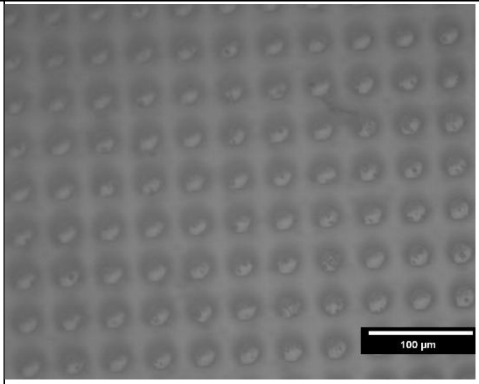	**dia20-dis30**	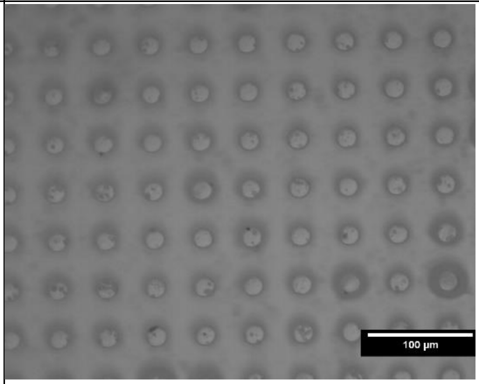
**dia25-dis25**	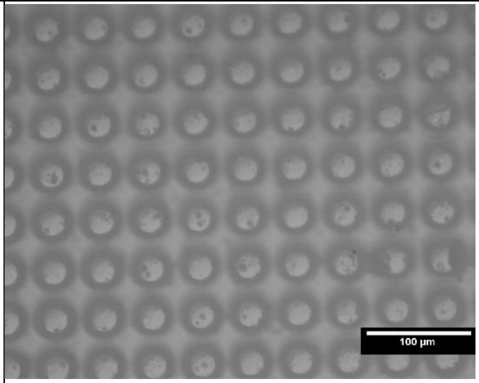	**dia40-dis40**	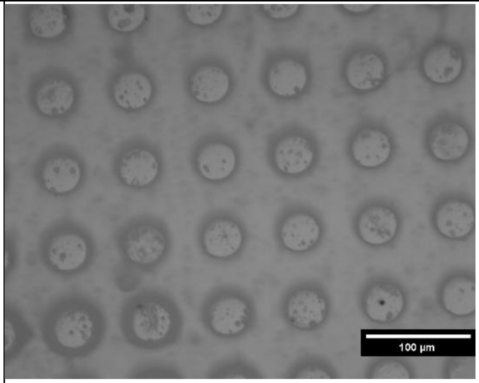

Figure S14: Optical microscope images of pillar type PLA patterns after CVD coating with DMDCS.

Table S4: Theoretical contact angles which were calculated by using Cassie-Baxter and Wenzel equations on the pillared PLA patterns after CVD coating with DMDCS in comparison with the experimental values.

Sample Name	Cassie θ (°)	Wenzel θ (°)	Measured θ_{app} (°) ± 1	Cassie-% Deviation	Wenzel-% Deviation
dia10-dis10	149	124	150	0,7	17,3
dia10-dis15	155	117	151	2,7	22,5
dia10-dis20	159	113	150	6,2	24,5
dia10-dis25	162	111	154	5,4	27,9
dia15-dis15	149	118	153	2,8	23,2
dia15-dis20	153	114	154	0,6	25,9
dia15-dis25	157	112	152	3,1	26,4
dia20-dis20	149	114	152	2,2	24,8
dia20-dis25	152	112	152	0,2	26,1
dia20-dis30	155	111	153	1,4	27,5
dia25-dis25	149	112	154	3,4	27,0
dia40-dis40	149	110	132	12,8	16,7

Sample Name	200x (After Rinse)	Sample Name	200x (After Rinse)
dia10-dis10	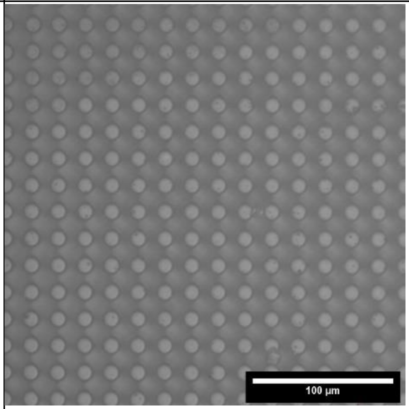	dia10-dis15	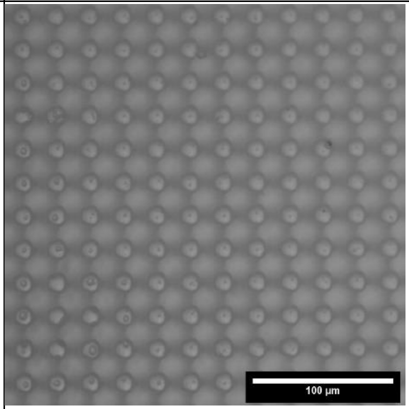
dia10-dis20	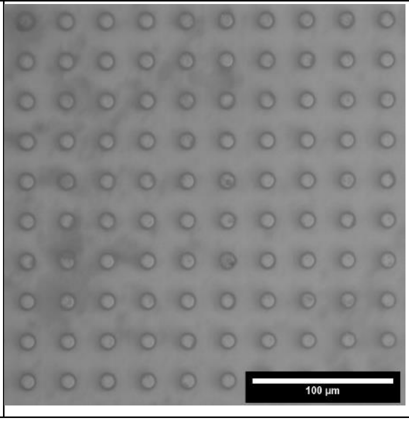	dia10-dis25	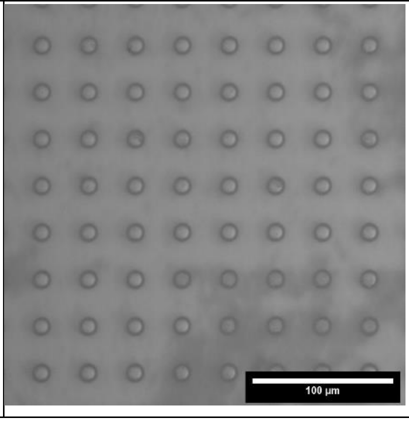

**dia15-dis15**	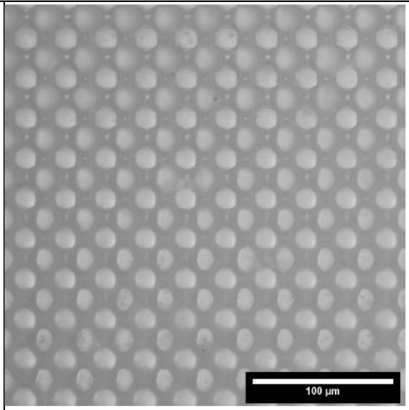	**dia15-dis20**	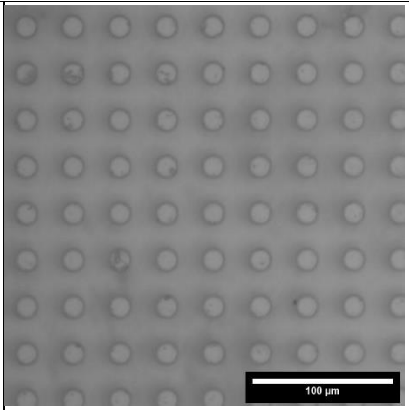
**dia15-dis25**	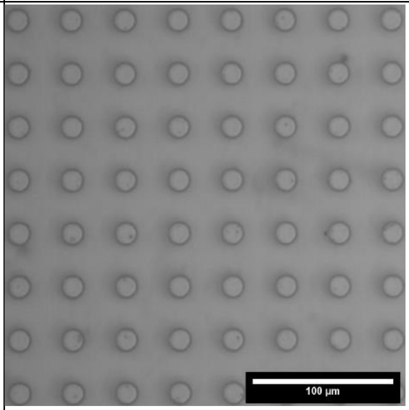	**dia20-dis20**	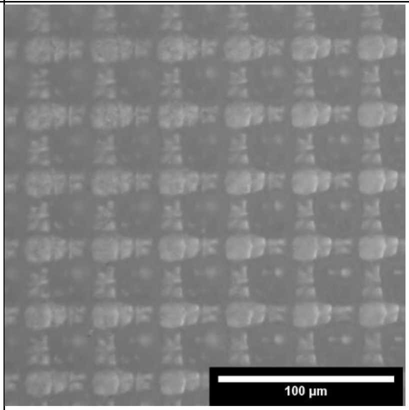
**dia20-dis25**	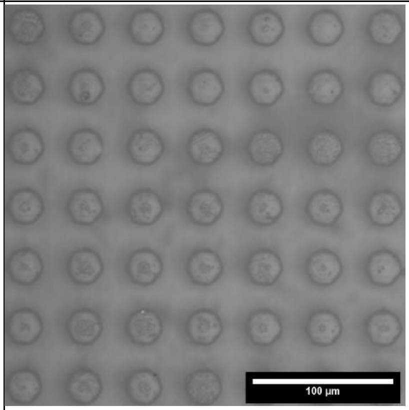	**dia20-dis30**	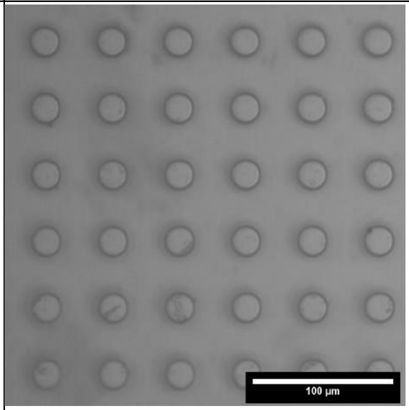
**dia25-dis25**	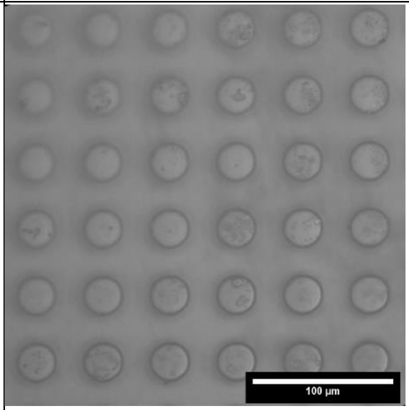	**dia40-dis40**	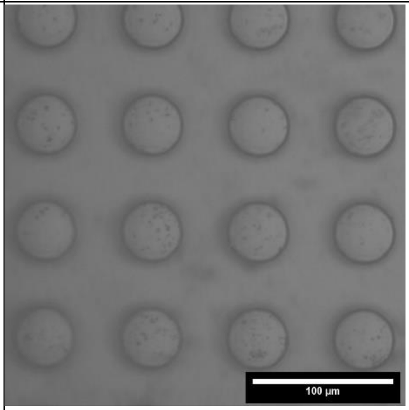

Figure S15. Optical microscope images of pillar type PLA patterns after CVD coating with NPTS.

Table S5. Theoretical contact angles which were calculated by using Cassie-Baxter and Wenzel equations on the pillared PLA patterns after CVD coating with NPTS in comparison with the experimental values.

Sample Name	Cassie θ (°)	Wenzel θ (°)	Measured θ_{app} (°) ± 1	Cassie-% Deviation	Wenzel-% Deviation
dia10-dis10	148	117	154	3,9	24,0
dia10-dis15	154	111	152	1,5	26,7
dia10-dis20	159	108	151	5,0	28,2
dia10-dis25	162	107	151	7,1	29,3
dia15-dis15	148	112	153	3,5	26,9
dia15-dis20	152	109	151	0,9	27,7
dia15-dis25	156	107	155	0,6	30,7
dia20-dis20	148	109	154	4,1	29,0
dia20-dis25	151	108	153	1,1	29,6
dia20-dis30	154	107	151	2,1	29,4
dia25-dis25	148	108	153	3,5	29,5
dia40-dis40	148	106	151	2,0	29,8

Sample Name	200x (After Rinse)	Sample Name	200x (After Rinse)
dia10-dis10	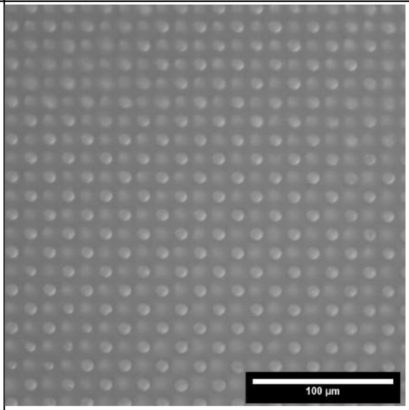	dia10-dis15	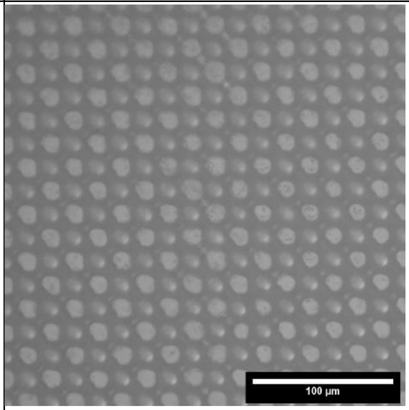
dia10-dis20	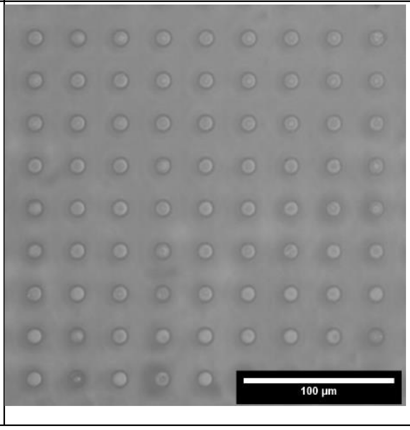	dia10-dis25	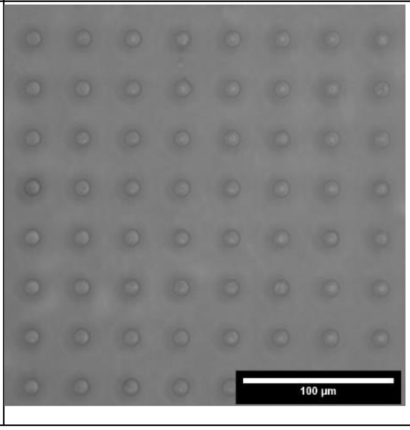

**dia15-dis15**	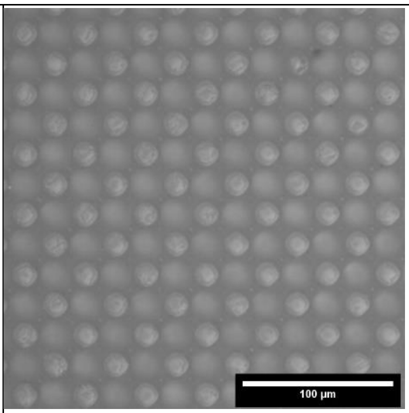	**dia15-dis20**	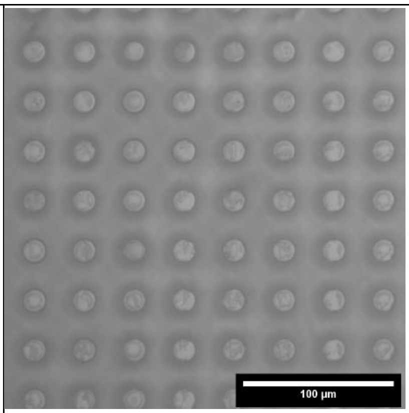
**dia15-dis25**	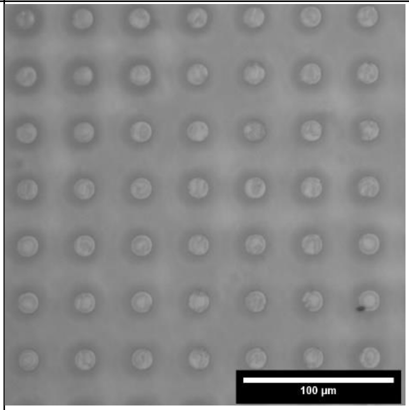	**dia20-dis20**	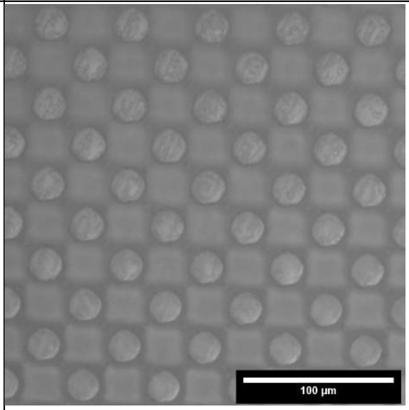
**dia20-dis25**	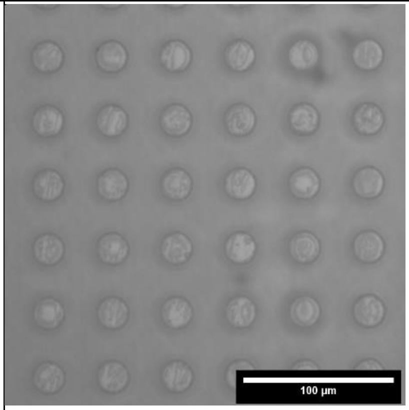	**dia20-dis30**	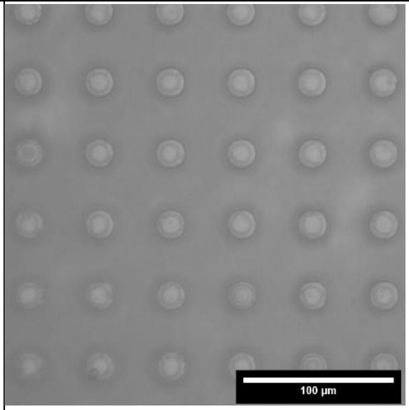
**dia25-dis25**	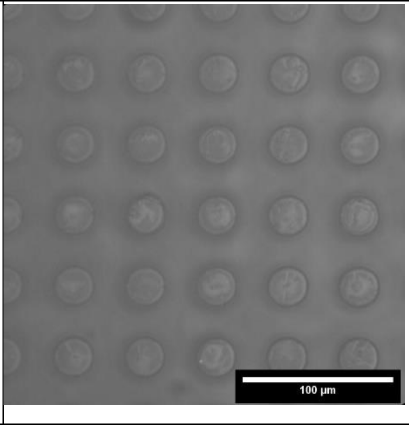	**dia40-dis40**	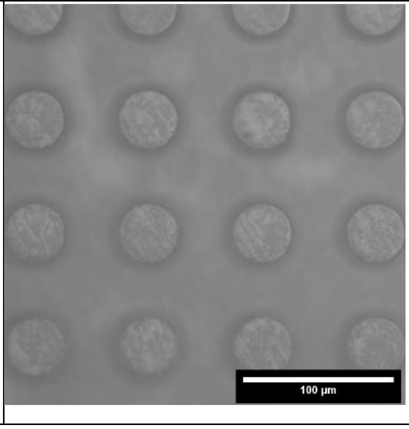

Figure S16. Optical microscope images of pillar type PLA patterns after CVD coating with TDFS.

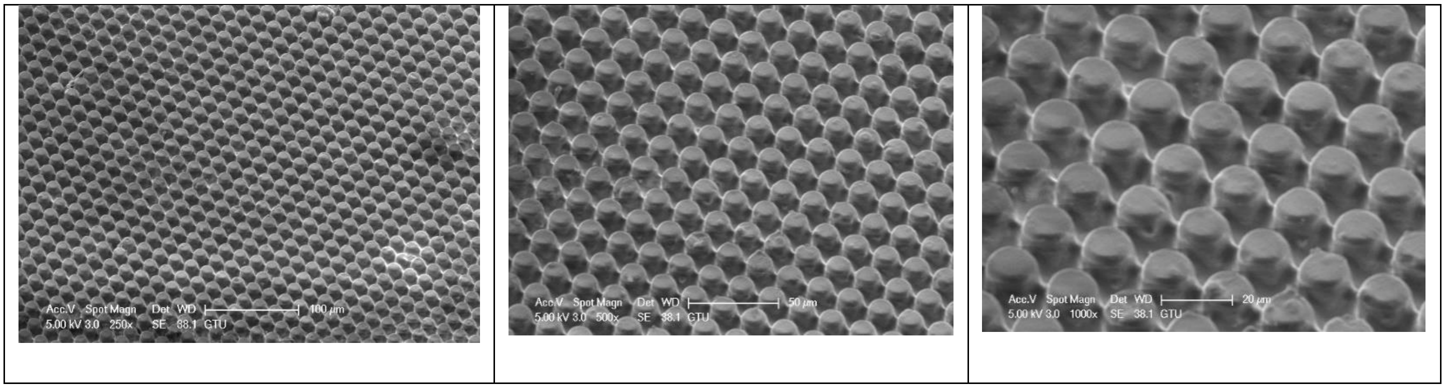

Figure S17. SEM images of PLA pattern after coating with DMDCS by using CVD method for (dia10-dis10) sample for 250, 500 and 1000x magnifications.

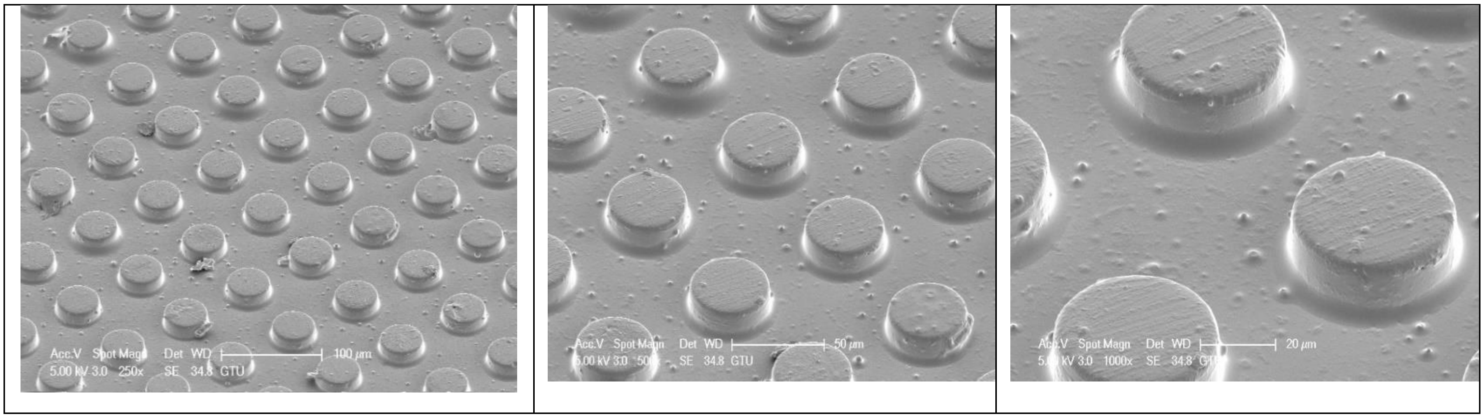

Figure S18. SEM images of PLA pattern after coating with DMDCS by using CVD method for (dia40-dis40) sample for 250, 500 and 1000x magnifications.

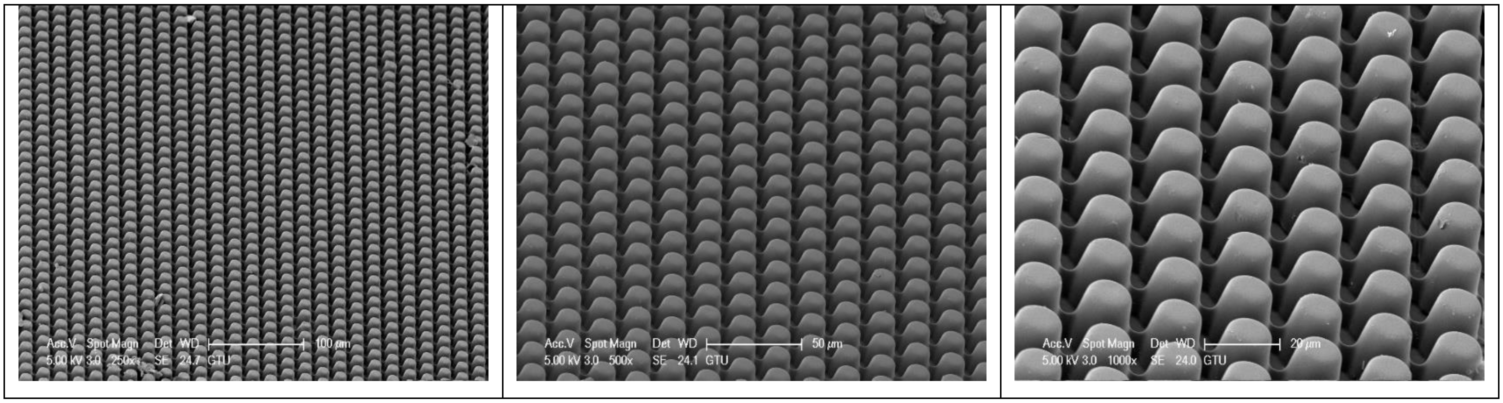

Figure S19: SEM images of PLA pattern after coating with TDFS by using CVD Method with pillar diameter=10 µm and spacing distance=10 µm for 250, 500 and 1000x magnifications.

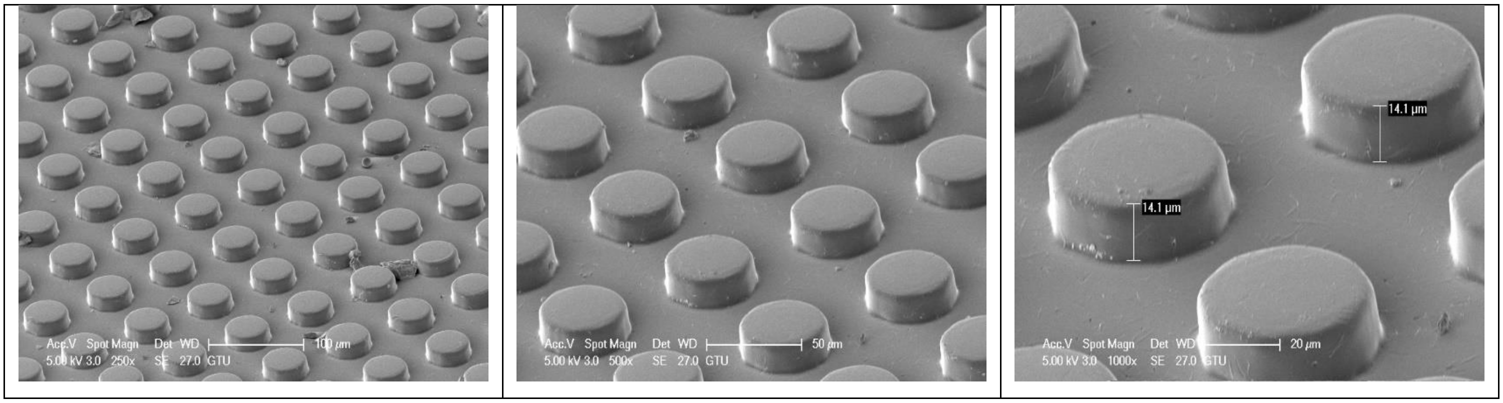

Figure S20: SEM images of PLA pattern after coating with TDFS by using CVD Method with pillar diameter=40 µm and spacing distance=40 µm for 250, 500 and 1000x magnifications.

CVD Silane	dia10-dis10	dia40-dis40
DMDCS	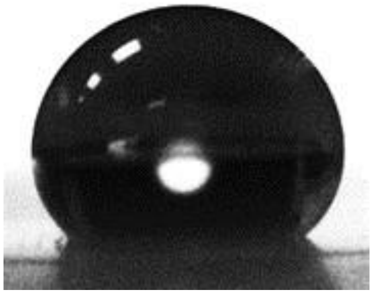	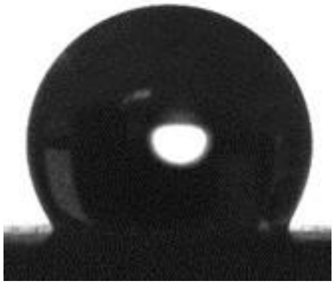
NPTS	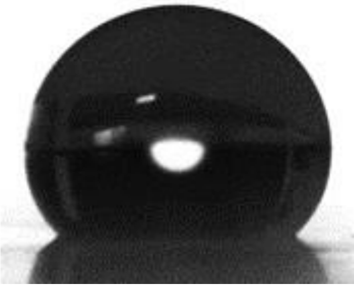	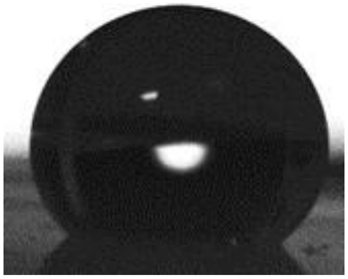
TDFS	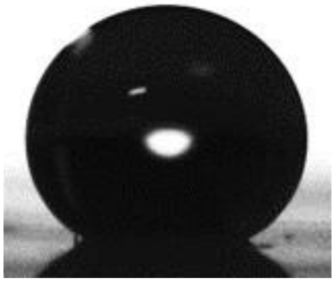	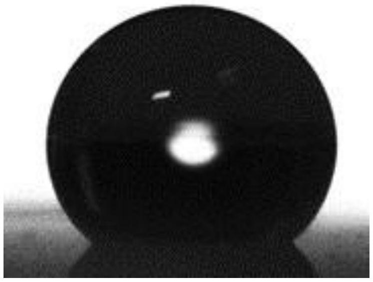

Figure S21. Indicative images of water droplet profiles on the micro-pillared PLA patterns after coating with DMDCS, NPTS and TDFS by CVD method.

Table S6: Theoretical contact angles which were calculated by using Cassie-Baxter and Wenzel equations on the pillared PLA patterns after CVD coating with TDFS in comparison with the experimental values.

Sample Name	Cassie θ (°)	Wenzel θ (°)	Measured θ_{app} (°) ± 1	Cassie-% Deviation	Wenzel-% Deviation
dia10-dis10	152	148	154	1,3	3,7
dia10-dis15	157	133	151	4,3	11,7
dia10-dis20	161	127	156	3,4	18,9
dia10-dis25	164	123	160	2,5	23,3
dia15-dis15	152	134	155	2,1	13,4
dia15-dis20	156	128	154	1,3	16,9
dia15-dis25	159	124	152	4,5	18,2
dia20-dis20	152	128	153	0,9	16,1
dia20-dis25	155	125	152	1,9	17,8
dia20-dis30	157	123	153	2,9	19,9
dia25-dis25	152	125	151	0,7	17,2
dia40-dis40	152	120	151	0,7	20,3

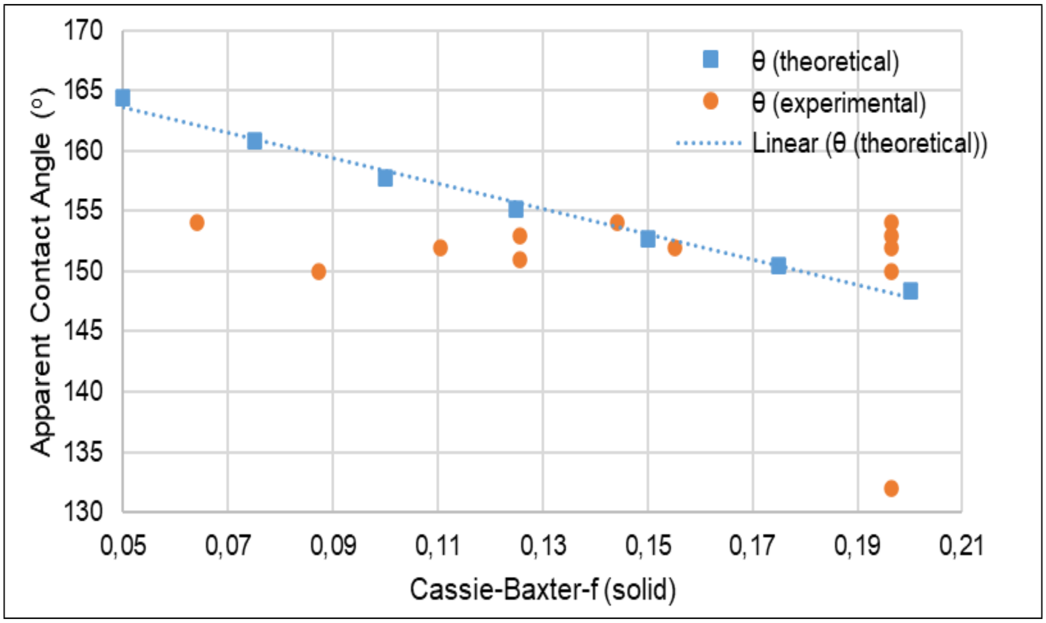

Figure S22. The deviation of apparent contact angles on the DMDCS coated PLA pillar type patterns from the Cassie-Baxter equation (blue dotted line).

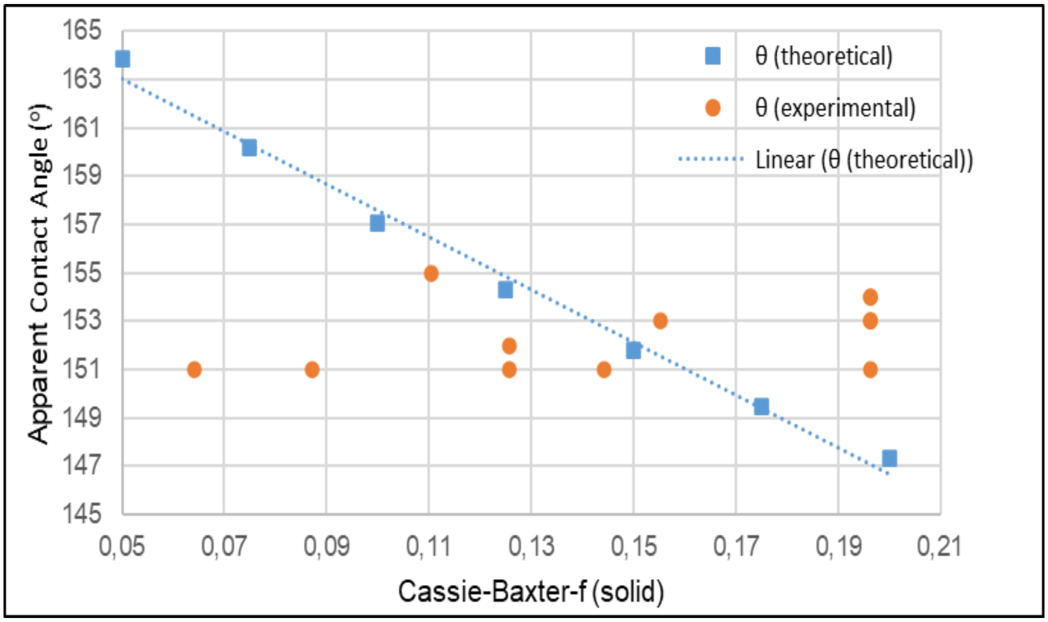

Figure S23. The deviation of apparent contact angles on the NPTS coated PLA pillar type patterns from the Cassie-Baxter equation (blue dotted line)

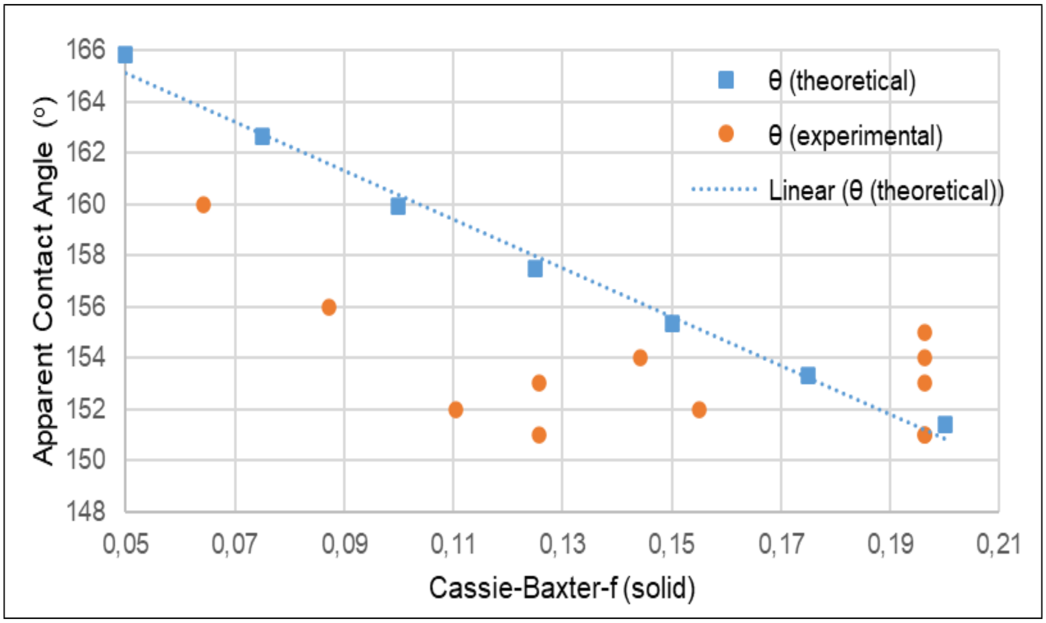

Figure S24. The deviation of apparent contact angles on the TDFS coated PLA pillar type patterns from the Cassie-Baxter equation (blue dotted line).
